# Short Linear Motifs (SLiMs) in “Core” RxLR Effectors of *Phytophthora parasitica* var. *nicotianae*: a Case of PpRxLR1 Effector

**DOI:** 10.1128/spectrum.01774-21

**Published:** 2022-04-11

**Authors:** Jane Chepsergon, Celiwe Innocentia Nxumalo, Brenda S. C. Salasini, Aquillah M. Kanzi, Lucy Novungayo Moleleki

**Affiliations:** a Department of Biochemistry, Genetics and Microbiology, Forestry and Agricultural Biotechnology Institute, University of Pretoria, Pretoria, South Africa; Broad Institute

**Keywords:** “core” RxLR effectors, SLiMs, *Phytophthora*, plant immunity, *Phytophthora* spp.

## Abstract

Oomycetes of the genus *Phytophthora* encompass several of the most successful plant pathogens described to date. The success of infection by *Phytophthora* species is attributed to the pathogens’ ability to secrete effector proteins that alter the host’s physiological processes. Structural analyses of effector proteins mainly from bacterial and viral pathogens have revealed the presence of intrinsically disordered regions that host short linear motifs (SLiMs). These motifs play important biological roles by facilitating protein-protein interactions as well as protein translocation. Nonetheless, SLiMs in *Phytophthora* species RxLR effectors have not been investigated previously and their roles remain unknown. Using a bioinformatics pipeline, we identified 333 candidate RxLR effectors in the strain INRA 310 of Phytophthora parasitica. Of these, 71 (21%) were also found to be present in 10 other genomes of P. parasitica, and hence, these were designated core RxLR effectors (CREs). Within the CRE sequences, the N terminus exhibited enrichment in intrinsically disordered regions compared to the C terminus, suggesting a potential role of disorder in effector translocation. Although the disorder content was reduced in the C-terminal regions, it is important to mention that most SLiMs were in this terminus. PpRxLR1 is one of the 71 CREs identified in this study, and its genes encode a 6-amino acid (aa)-long SLiM at the C terminus. We showed that PpRxLR1 interacts with several host proteins that are implicated in defense. Structural analysis of this effector using homology modeling revealed the presence of potential ligand-binding sites. Among key residues that were predicted to be crucial for ligand binding, L^102^ and Y^106^ were of interest since they form part of the 6-aa-long PpRxLR1 SLiM. *In silico* substitution of these two residues to alanine was predicted to have a significant effect on both the function and the structure of PpRxLR1 effector. Molecular docking simulations revealed possible interactions between PpRxLR1 effector and ubiquitin-associated proteins. The ubiquitin-like SLiM carried in this effector was shown to be a potential mediator of these interactions. Further studies are required to validate and elucidate the underlying molecular mechanism of action.

**IMPORTANCE** The continuous gain and loss of RxLR effectors makes the control of *Phytophthora* spp. difficult. Therefore, in this study, we endeavored to identify RxLR effectors that are highly conserved among species, also known as “core” RxLR effectors (CREs). We reason that these highly conserved effectors target conserved proteins or processes; thus, they can be harnessed in breeding for durable resistance in plants. To further understand the mechanisms of action of CREs, structural dissection of these proteins is crucial. Intrinsically disordered regions (IDRs) that do not adopt a fixed, three-dimensional fold carry short linear motifs (SLiMs) that mediate biological functions of proteins. The presence and potential role of these SLiMs in CREs of *Phytophthora* spp. have been overlooked. To our knowledge, we have effectively identified CREs as well as SLiMs with the potential of promoting effector virulence. Together, this work has advanced our comprehension of *Phytophthora* RxLR effector function and may facilitate the development of innovative and effective control strategies.

## INTRODUCTION

Pathogens and plants are locked in a molecular arms race (1–3). The success of these pathogens is attributed largely to their ability to secrete effectors (4, 5). Effectors are a heterogeneous group of proteins that are secreted by pathogens to manipulate the host immunity and facilitate disease development. Structural diversity in effector proteins is a critical factor that drives the coevolutionary interactions between plants and pathogens (6, 7). Oomycetes secrete hundreds of effector proteins into the host cells to modulate cellular processes (8–11). Among these effectors are the RxLR proteins, named after the sequence motif arginine-any amino acid-leucine-arginine (12, 13). The majority of RxLR effectors are upregulated during infection, and those expressed during the biotrophic phase have been targeted for further characterization (14–16). However, necrotrophs like *Pythium* have also been reported to harbor a repertoire of RxLR effectors (17). Therefore, it could be reasoned that RxLR effectors comprise a superfamily of effectors in oomycetes. RxLR effectors have modular structures, where the N terminus contains a signal peptide, followed by the signature RxLR motif, which functions in effector translocation into host cells, while the C-terminal domains carry the effector activity (18, 19). Nearly half of RxLR effectors (44%) from *Phytophthora* spp. and 26% from Hyaloperonospora arabidopsidis have a highly conserved W and Y motif at the C terminus (13, 20, 21).

For decades, the biological functionality of proteins, including RxLR effectors, has been strongly associated with their unique 3D structures (20, 22, 23). Nonetheless, recent studies have revealed the existence of “hybrid” proteins (24, 25). These are proteins that consist of ordered domains as well as intrinsically disordered regions (IDRs) (24, 26). In fact, a good portion of known protein sequences consist of IDRs that do not fold into well-defined or stable 3D structures. For instance, prediction studies have estimated that approximately 14% of the bacterial proteome, as well as 44 to 54% of both eukaryote and virus proteomes, is disordered; this is commonly known as “dark proteome” (27, 28). Several studies have clearly shown that these IDRs are not only abundant but also actively involved in numerous cellular/biological processes such as signaling and recognition (29, 30), gene regulation (31, 32), and protein degradation (33). The long-standing conundrum has been the question of how these IDRs perform such specific functions without a well-defined structure. Important discoveries over the past decade have revealed the presence of high-accessibility sites within the disordered proteins (30, 34). The existence of these sites simplifies posttranslational modifications of IDRs, allowing for a simple way to modulate their biological functions (22). Some of the best-studied IDRs are short linear motifs (SLiMs). SLiMs are short stretches of protein sequences, about 3 to 10 amino acids (aa) long. They are crucial for biological processes because they facilitate protein-protein interactions (22, 35). Consequently, SLiMs are ideal elements to tune functionality in eukaryotic regulatory proteins. In addition, (i) they are located natively in the disordered protein regions and, if within folded domains, tend to reside in accessible loops (33), and (ii) they demonstrate high levels of plasticity in that motifs may appear or disappear as a result of single point mutations (36).

The concept of IDRs and SLiMs has been extensively studied in proteomes of viruses (37, 38) as well as effector proteins of bacterial pathogens (39, 40). Recently, the virulence activity of effector proteins of phytopathogenic oomycetes, mainly the RxLR effector proteins, has been associated with IDRs carried in these effectors (41, 42). Although several conserved or “core” RxLR effectors (CRE) of oomycetes have been identified, only a few of these have been functionally characterized, hence making it difficult to have a consensus on the term CREs. In a recent review, CREs were defined as effectors that are conserved among strains of either a pathogen or different pathogen species, with the potential of playing a virulence role during infection process (43). These effectors are of importance since they are potential targets for breeding for durable resistance in plants (44, 45). To the best of our knowledge, there are no studies that have been done to identify the abundance of IDRs together with SLiMs in conserved CREs. In this work, we predicted a total of 71 CREs in Phytophthora parasitica INRA 310. The majority of these CREs contained IDRs as well as SLiMs.

## RESULTS

### A small portion of *P. parasitica* proteome is secreted.

**FIG 1:**
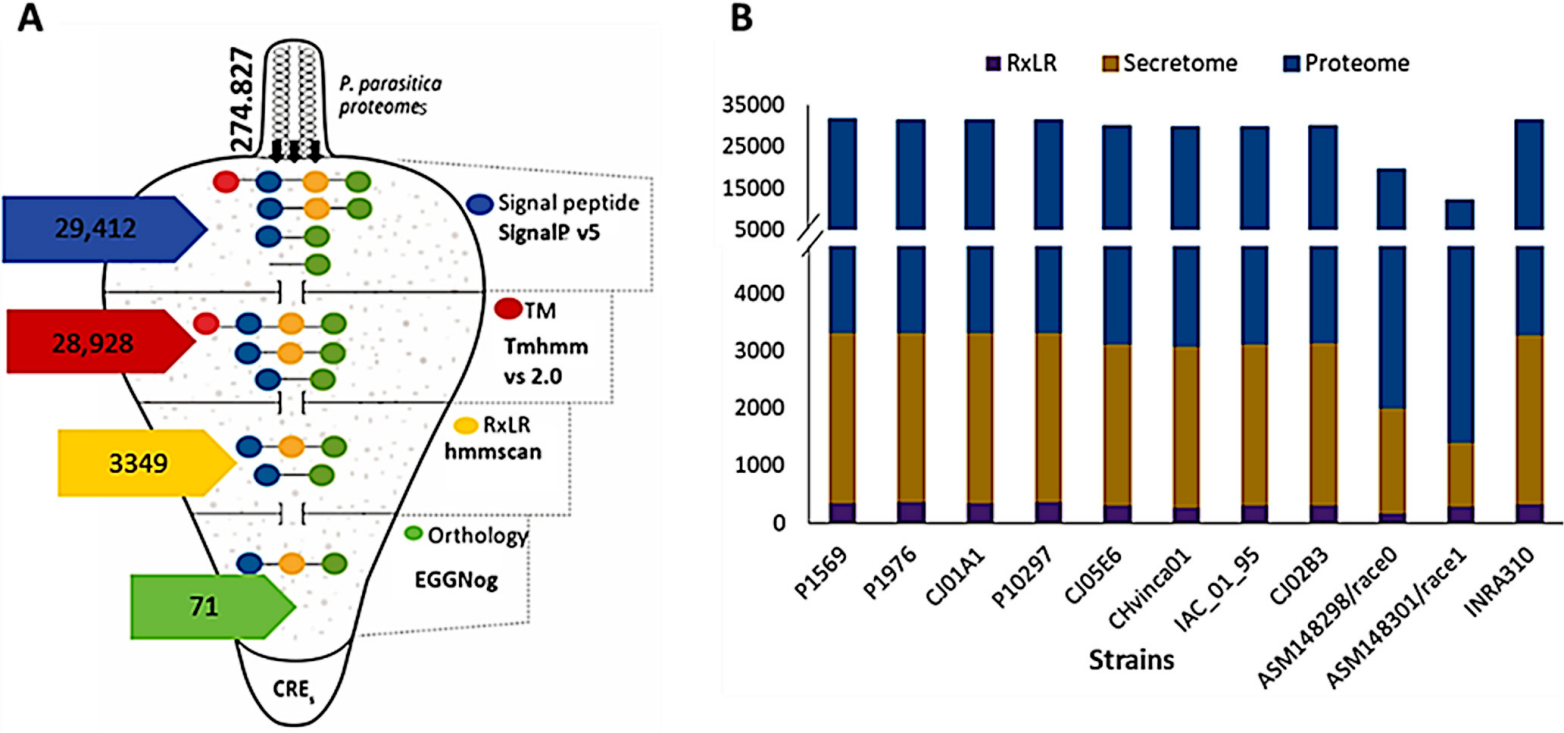
Number of proteins obtained in *P. parasitica* predicted secretome. (A) Pipeline designed to predict and compare candidate RxLR effectors (CREs) in the genomes of *P. parasitica*. The pipeline was implemented to predict signal peptide (SP; designated by a blue filled circle)-containing proteins and to exclude those with N-terminal transmembrane domains (TMs; in red). The signature motif RxLR (yellow) was identified using a hidden Markov model (HMM) scan. Orthology analysis using EGGNog mapper was conducted to retain RxLR effectors (green) that are conserved within the 11 strains of *P. parasitica*, also known as core RxLR effectors. (B) The number of the predicted secretomes (orange) relative to the assessed proteomes (blue) of *P. parasitica* strains as well as estimated number of RxLR effector proteins (purple). *Phytophthora parasitica* RxLR effector proteins are located largely in gene-sparse regions (GSRs) of the genome.

The success of phytopathogenic oomycetes, like *Phytophthora* spp., is augmented by their ability to secrete effector proteins that enable host colonization (46). Taking advantage of the availability of sequenced genomes of 11 strains of *P. parasitica*, we employed an *in silico* prediction pipeline (Fig. 1A) to estimate the number of proteins secreted by this pathogen (see Materials and Methods). Our data set consisted of predicted proteomes (complete set of proteins as expressed by a genome) of 11 strains of *P. parasitica* var. *nicotianae*. In total, the proteomes of these 11 pathogens amount to 274,827 proteins. Of these, the predicted secretomes (set of proteins potentially soluble and secreted) ranged between 1,107 and 2,959 proteins, which comprise approximately 6.3 to 26.6% of the total proteomes (Fig. 1B). We further determined the proportion of RxLR effectors in the secretomes of the 11 strains of *P. parasitica* (Fig. 1B). The total number of potentially secreted RxLR effectors in these strains ranged between 165 and 358. Variations in the number of RxLR effectors among the 11 strains was shown to be independent of the genome size (Fig. S1). This seems to be the trend in *Phytophthora* spp. For instance, Phytophthora infestans is a narrow-host-range pathogen with the biggest genome size (240 Mb) and the highest number of RxLR effectors (563) (12). Phytophthora ramorum and Phytophthora sojae, also with narrow host ranges, have significantly smaller genomes of 65 Mb and 95 Mb, respectively, but present a relatively high number of RxLR effectors (350) (47). On the other hand, Phytophthora cactorum is a wide-host-range pathogen with a genome size of 121.5 Mb but carries only 199 RxLR effectors (48), suggesting that the number of CREs present in a genome might be related more to the source host rather than the genome size.

### A subset of RxLR effectors in *P. parasitica* is conserved.

Recent studies on the potential virulence of CREs in *P. parasitica* support their pivotal role in virulence activity of this pathogen (15, 49, 50). Since an arsenal of RxLR effectors are predicted to be secreted by *P. parasitica* (Fig. 1A and B), we set out to determine the occurrence of core RxLR effectors across the 11 strains of the species. To this end, we scanned the presence of homologs of the 333 RxLR effectors from INRA 310 strain in the data sets of the other 10 strains using the EggNOG orthology tool with default parameters (Fig. 1A). The orthology analysis revealed that a total of 71 out of 333 (21%) RxLR effectors are shared in all the 11 isolates (Table S1). These sequences are referred to here as core RxLR effectors (CREs) of *P. parasitica*.

**FIG 2:**
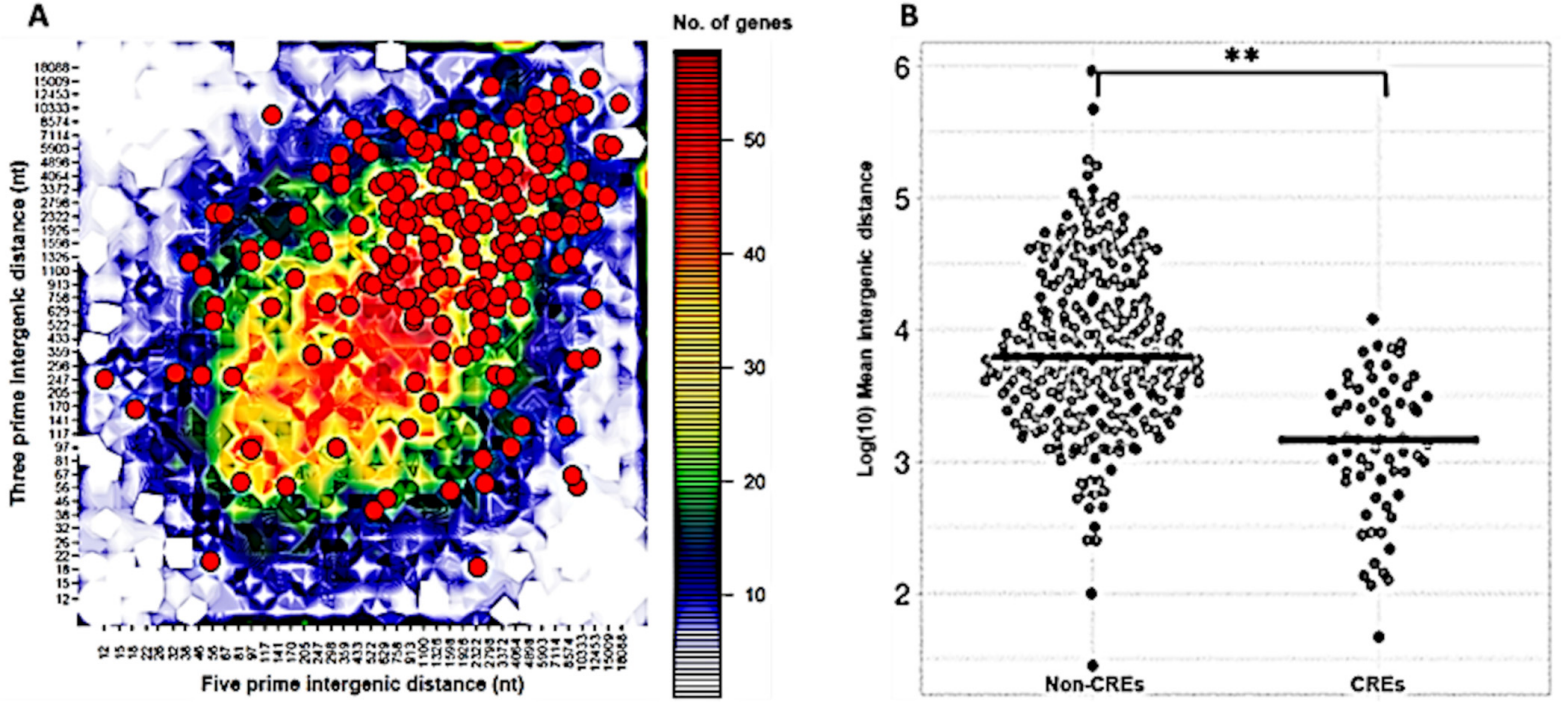
Distribution of *P. parasitica* RxLR effector genes according to the length of their FIRs. (A) Genome architecture of *P. parasitica* isolate INRA 310 with the 333 candidate RxLR effectors bin plotted according to gene density using 5′ and 3′ intergenic border lengths. The color of each bin represents the number of genes, i.e., 40 to 50 genes (red),20 to 30 (green), and 10 (blue). RxLR effectors, shown in red circles, tend to be distributed across both gene-sparse (blue-green) and gene-dense (yellow-red) regions of the genome. (B) Comparisons between conserved (CREs) and nonconserved RxLR genes. Non-CREs have a significantly longer mean intergenic distance than CREs. **, *P* < 0.01.

So far in this study, we have shown that only a small portion of the RxLR effector genes, 71/333 (21%), are conserved across the genomes of *P. parasitica* strains, probably because these genes are evolving rapidly. In addition, *Phytophthora* species’ genomes have been classified as bipartite architecture with the gene-sparse, repeat-rich compartment serving as a cradle for adaptive evolution (51). We were therefore motivated to investigate the genomic distribution of candidate RxLR effector-encoding genes in *P. parasitica* strain INRA 310. The length of the flanking intergenic region (FIR) between neighboring genes provides a measurement of the local distribution of gene density. This can be plotted into a two-dimensional graph based on the length of intergenic regions between the 5′ and 3′ ends of neighboring genes (Fig. 2A). Continuous distribution of RxLR effectors (red dots) across both gene-sparse (blue-green) and gene-dense regions (yellow-red) was observed. We further show that compared to those of nonconserved RxLR effector genes, the 71 core RxLR effector genes (CREs) identified in the genome of *P. parasitica* recorded significantly shorter intergenic distances (Fig. 2B). Since effector genes, including those encoding RxLR effectors, have been found to reside in close proximity to transposable elements (TEs) (52), the increased intergenic distances of non-CRE genes in Fig. 2B could be due to the insertion and expansion of TEs.

### Intrinsic disorder is a feature of CREs in *P. parasitica*.

**FIG 3:**
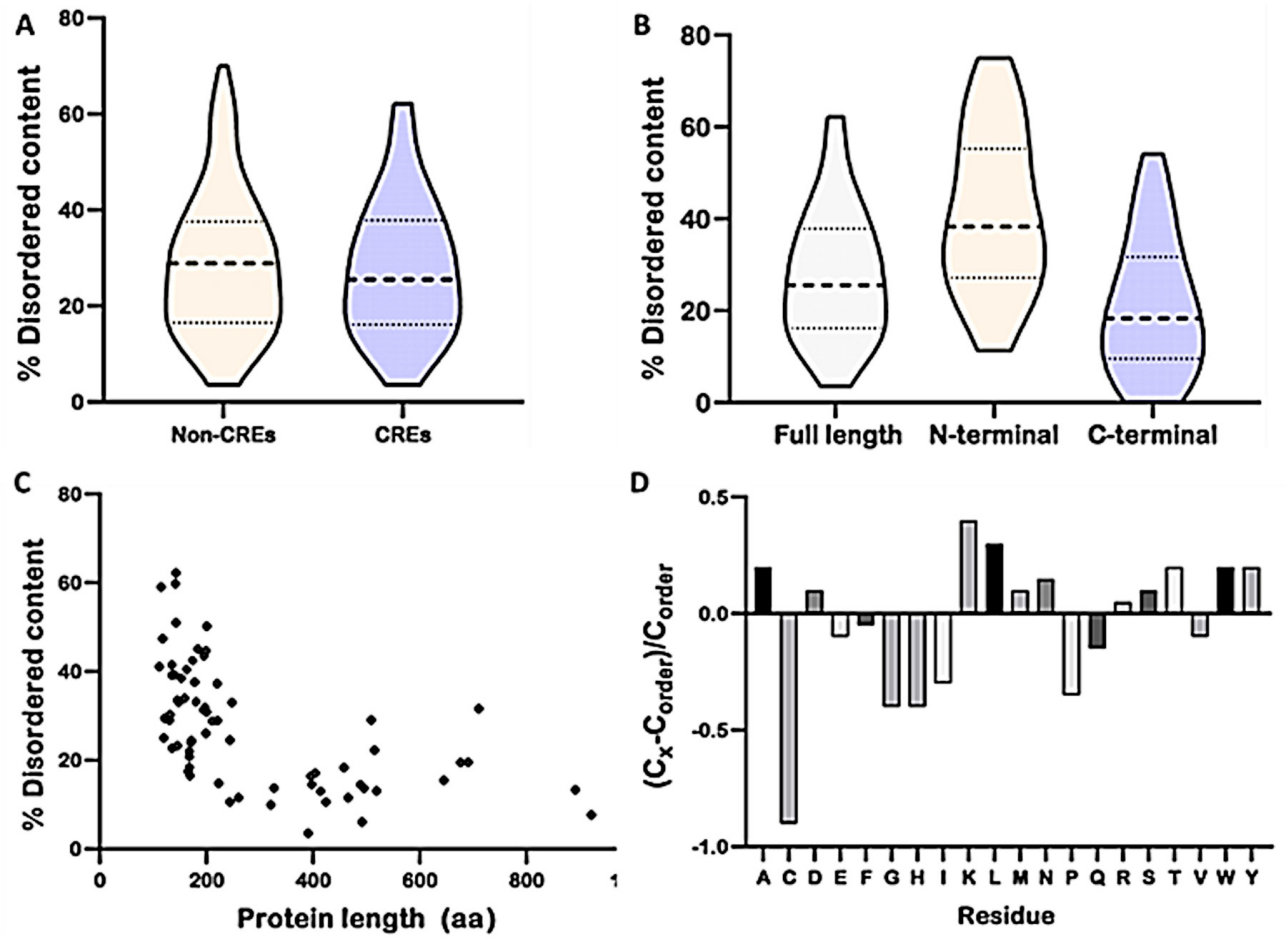
Disordered content of *P. parasitica* RxLR effectors. (A) Comparison of disordered content between core RxLR effectors (CREs) and non-CRE proteins. (B) Distribution of disorder content in CRE protein sequences. The violin plots show medians (bold dotted line), 25% and 75% quartile boundaries (light dotted line), and total ranges (whiskers) of all the proteins. (C) Disordered segment length relative to the protein length. Nonlinear negative correlation was recorded (*r* = −0.61; *P* < 0.001). (D) Amino acid composition comparison between conserved RxLR proteins and ordered proteins using Composition Profiler. The fractional difference was calculated as (*C*_x_ − *C*_order_)/*C*_order_, where *C*_x_ was the average amino acid composition in the conserved RxLR data set while *C*_order_ was the value for the control set of ordered proteins from PDB Select 25. Positive bars correspond to residues found more abundantly in RxLR proteins, whereas negative bars correspond to residues depleted in RxLR effector proteins.

Thus far, we have established that the genomes of *P. parasitica* carry an arsenal of CREs which could potentially be of importance during disease development. Recently, RxLR effectors of oomycetes have been shown to possess IDRs, which are potentially key players in the biological activity of these effectors (41, 42). We were therefore motivated to investigate the occurrence of IDRs in CREs of *P. parasitica*. Overall, the difference in disordered content between CREs and nonconserved RxLR effectors in *P. parasitica* was not significant (Fig. 3A). The mean disorder content in each predicted conserved RxLR protein ranged between 3 and 62% with an average of 26% (Fig. 3B). To explore the distribution of disorder content throughout the RxLR protein sequence, we analyzed predicted CREs in their N- and C-terminal regions. The N terminus comprised a signal peptide (SP) and a RxLR-dEER region, while the remaining effector domain region formed part of the C terminus. The N-terminal regions exhibit 38% of disordered content, while the C terminus had 18% (Fig. 3B). Therefore, it is likely that IDRs in CREs of *P. parasitica* are implicated in effector translocation into the host cell.

We further ascertained whether there is any relationship between disordered content in CREs and protein size. Protein lengths of the 71 CREs in *P. parasitica* ranged between 112 and 922 residues, with an average disordered segment length of 14.4% (Fig. 3B) representing a minimum of 3% and a maximum of 40% length of disordered segment. By looking at the association between protein length and the length of their respective disordered regions, we conducted Spearman’s rank correlation where a negative correlation (*r* = −0.61; *P* < 0.001) was observed (Fig. 3C). This suggests that the disordered content could be largely associated with short proteins rather than long protein sequences.

### Amino acid composition of CREs in IDRs.

Disordered protein regions are defined by the nature of the amino acids in these regions. Therefore, investigating the amino acid composition biases could provide useful insights on the nature of CREs. It is known that disordered and ordered protein regions have significantly different amino acid compositions (26). With this in mind, we employed the Composition Profiler tool (http://www.cprofiler.org/) to analyze amino acid composition biases of our CRE data set in *P. parasitica*. As a rule of thumb, IDRs lack ordered structure because of specific amino acid biases, as they are typically depleted in order-promoting residues (Cys, Trp, Tyr, Phe Ile, Leu, Val, and Asn) and enriched in disorder-promoting residues (Pro, Arg, Gly, Gln, Ser, Glu, Lys, and Ala) (53). We compared our CRE data set with globular proteins from the protein data bank (PDB) Select 25 (54). As expected, we observed depletion in hydrophobic and aromatic (order-promoting) residues (Ile, Met, Leu, Val, Asn, Cys Phe, Trp, and Tyr) and conspicuous presence of structure-breaking residues (Pro and Gly, Ala, Arg, and Ser) (Fig. 3D).

### SLiMs are found in the C-terminal regions of CREs.

**FIG 4:**
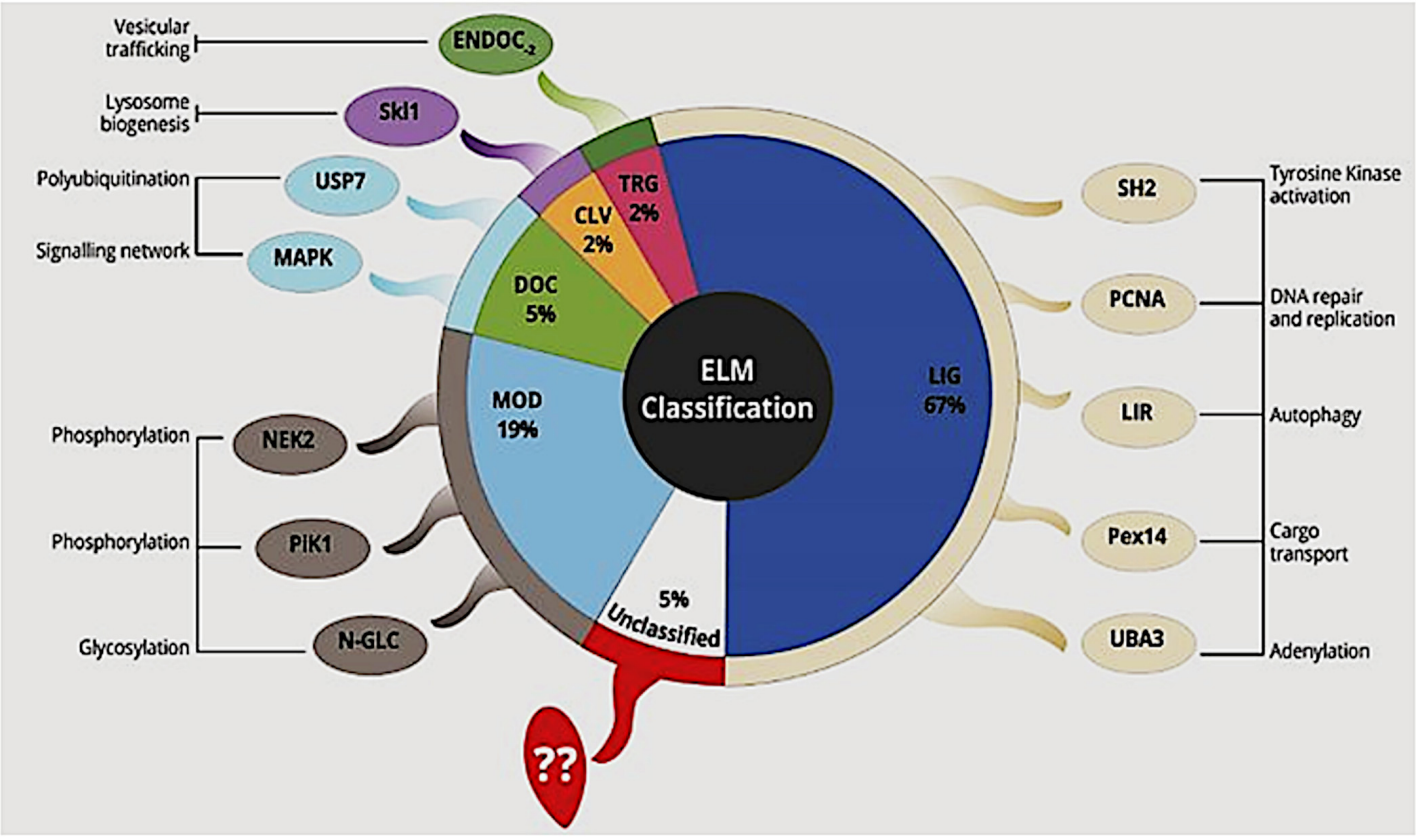
Proportion of SLiM class type in the ELM database found in CREs. The majority (66.7%) of the SLiMs found in CREs were classified as ligand-binding sites. Posttranslational modification sites, MOD (19%); docking, DOC (5%); targeting, TRG (2.4%); proteolytic cleavage site, CLV (2.4%). Five percent were not found in the ELM database. Potential ligand-binding sites of CREs target crucial processes, including adenylation, kinase activation, and autophagy as well as vesicle trafficking. Posttranscriptional modification, mainly phosphorylation and glycosylation, are also some of the potential roles of CRE SLiMs.

We have shown that approximately 21% of the disordered content is found in the C-terminal region of CREs (Fig. 3B). Since the C terminus is the main effector domain that carries out the biological activity of RxLR effectors, we hypothesized that these IDRs could be hosting active binding domains/motifs of CREs. It is well-known that SLiMs are functional stretches of protein sequences, with about 80% of these being located within the IDRs (55). Consequently, we endeavored to establish whether SLiMs are present/enriched in the C terminus of CREs of *P. parasitica*. To achieve this, we performed an *in silico* prediction of potential SLiMs using ANCHOR tool (56). The prediction was guided by three rules (56, 57): (i) the potential SLiM residues must be found along the IDRs and any globular domain is filtered out, (ii) predicted residues should lack the ability to form favorable contacts that may lead to folding and hence formation of well-defined structure, and (iii) the residues must have the potential to form favorable interactions with globular proteins upon binding. Using these criteria, our findings revealed that 27 of CREs (38%) have potential SLiMs (Fig. S2). We further assessed whether these predicted SLiMs have any known biological activity within the host cell. To achieve this, we employed the Eukaryotic Linear Motif resource (ELM) (58). ELM assigns motif classes to one of six functional categories: ligand-binding sites (LIG) mainly for protein-protein interactions, targeting (TRG) for subcellular localization, docking (DOC), degradation (DEG), posttranslational modification sites (MOD), or proteolytic cleavage site (CLV) motifs. The results obtained from CREs’ SLiMs satisfied this classification (Fig. 4). It is worth noting that about 44% of CRE sequences encoded more than one SLiM. A study needs to be conducted to determine whether this is a strategy that *P. parasitica* employs to target more than one protein/process.

### PpRxLR1 is a bona fide member of RxLR effector family.

**FIG 5:**
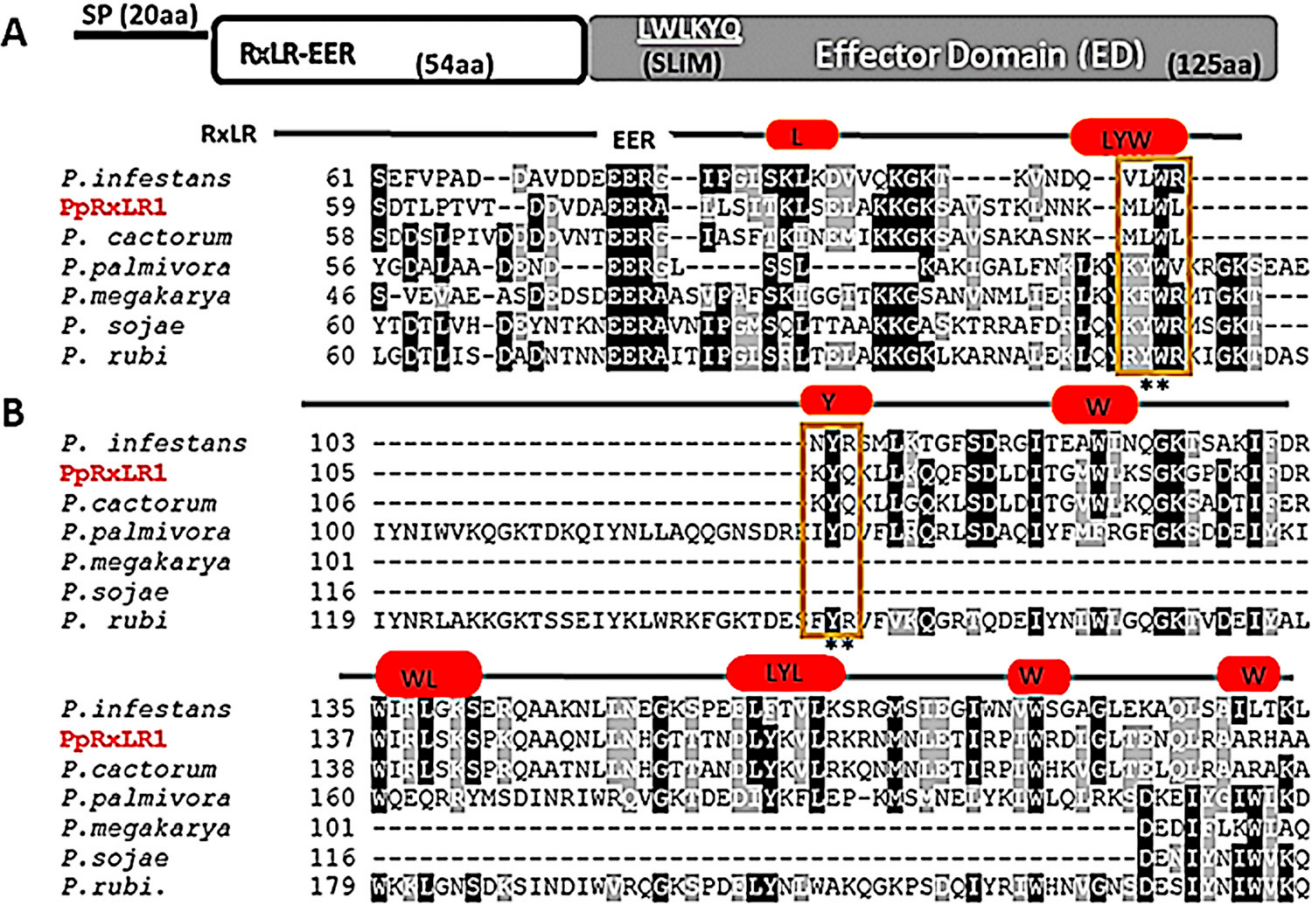
(A) Schematic representation of the PpRxLR1 effector, depicting the predicted amino acid sequence length of the signal peptide (SP), host‐translocation motif‐containing domain (RxLR‐EER), and effector domain (ED) that contains a putative SLiM (underlined). (B) Sequence alignment of PpRxLR1 effector and its orthologs in other six species of *Phytophthora*. PpRxLR1 shares sequence identity with homologues of other *Phytophthora* species. The C-terminal region of these sequences is characterized by LWY motifs (red boxes). Predicted SLiM (outlined in yellow) carried two evolutionary conserved residues (**), L^102^ and Y^106^. The dashes (–) indicate missing residues.

Of the 71 CREs that we identified, PpRxLR1 effector (Fig. 5A) was selected for further functional characterization based on the following reasons: (i) it is highly expressed after 30 h of *P. parasitica* infection (59), (ii) it has not yet been functionally characterized, and(iii) it is widely conserved among strains of *P. parasitica* (Fig. 5B). PpRxLR1 gene encodes a protein of 199 amino acids with a molecular mass of 22.09 kDa. It contains a predicted 20‐amino‐acid‐long N‐terminal signal peptide followed by an RxLR‐dEER motif starting at position 55 to 74. The remaining part of this protein forms a putative functional C-terminal domain also known as the effector domain (ED). Anchor tool (http://anchor.elte.hu/) and MoRFpred (http://biomine.cs.vcu.edu/servers/MoRFpred/) predictions revealed a putative SLiM site at the C‐terminal end of PpRxLR1 carrying six amino acids between Leu^102^ and Gln^107^ (Fig. 5A). Classification of this motif using Eukaryotic Linear Motif (ELM) revealed its potential as a ubiquitin-associated (UBA) motif (Table S2) that is implicated in the NEDD8 cascade, regulating cullin neddylation. Cullins are part of multisubunit cullin-based E3s (CRLs), playing an important role in substrate ubiquitination and consequently regulated protein degradation (60). Sequence alignment of PpRxLR1 and its orthologs showed high similarities in the N-terminal region (EER) but had a less-conserved C terminus, which is characterized by repeats of LWY motifs (Fig. 5B). These LWY motifs have been shown to play both structural and biochemical roles in the RxLR effectors (17, 61).

### PpRxLR1 has the potential to interact with various host proteins.

**FIG 6:**
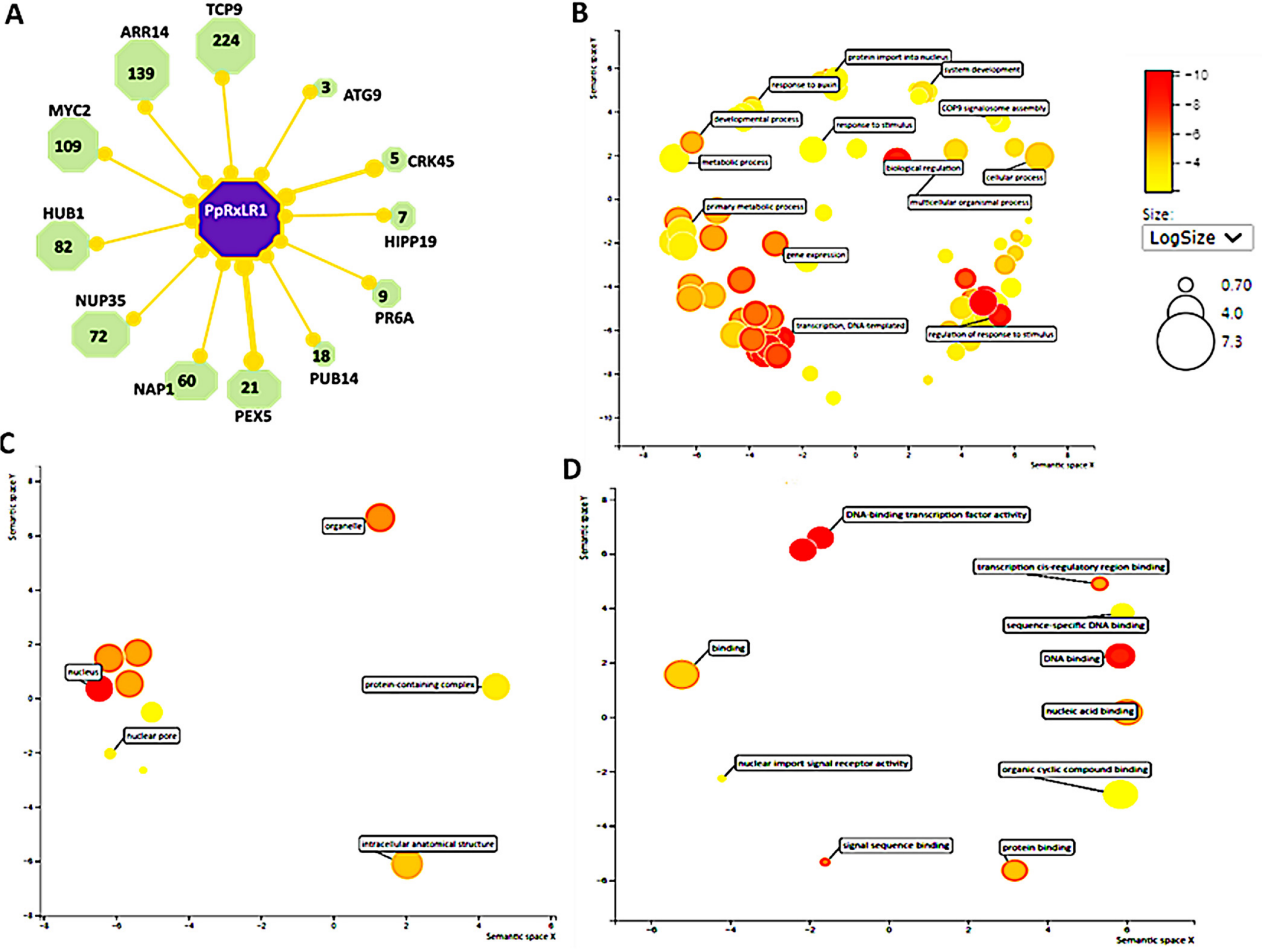
(A) Graphical representation of the interactomic data depicts PpRxLR1 (purple) possible interaction with host proteins as nodes (green) interconnected by edges (orange lines). PpRxLR1 interacts with various *A. thaliana* proteins (only 12 shown for better visualization). These include transcription factors regulators (TCP9), MYC-related transcriptional activator (MYC2), histone mono-ubiquitination (HUB1), nuclear pore complex protein (NUP35), nucleosome assembly protein (NAP1), peroxisomal targeting signal (PEX5), plant U-box type E3 ubiquitin ligases (PUB14), heavy metal-associated isoprenylated plant protein (HIPP19), cysteine-rich receptor-like protein kinase (CRK45), and autophagy protein (ATG9). The thickness of the connecting edges indicates the level of confidence: narrow edges represent physical interaction detected by only one technique, whereas thick edges indicate that the interaction has been detected by at least two independent techniques (e.g., coimmunoprecipitation and pulldown assays, Y2H). (B, C, and D) Gene Ontology (GO) bubble plot constructed using REVIGO for biological processes (B), cellular components (C), and molecular functions GO terms (D). Settings used for REVIGO program were as follows: database, Bos taurus. semantic similarity, 0.7 (medium). Semantic similarity measure, SimRel. Colors indicate the *P* value of the enriched GO terms, while the size of the bubbles indicates the frequency of the GO terms.

Since PpRxLR1 is a potential “core” effector, we hypothesized that *P. parasitica* uses this effector to target the most influential proteins in their host network. To test this concept, PpRxLR1 sequence was submitted to the AraPathogen predictor that predicts protein-protein interactions (PPIs) between Arabidopsis thaliana and pathogens based on sequence and A. thaliana intraspecies PPI network (InterSPPI) (62). We show that PpRxLR1 effector has the potential to interact with 161 *A. thaliana* proteins (Table S3), forming a fuzzy interaction network (Fig. S3). Therefore, for better visualization, only 12 proteins were randomly selected to generate the interaction network using IntAct network on Cytoscape (Fig. 6A). We focused on two main network topology parameters: degree and betweenness centrality. The degree of a protein represents the number of proteins that it interacts with, while betweenness centrality of a protein is the fraction of all shortest paths connecting two proteins from the network that pass through it.

Our findings reveal that PpRxLR1 effector interactors showed a high degree of host protein interaction. For example, the effector has the potential to interact with transcription factor (TCP) and autophagy-related proteins (ATG), which form networks with 224 and 3 host proteins, respectively. It is therefore likely that core effectors like PpRxLR1 have the potential to interact with more highly connected and central *A. thaliana* proteins, and this might imply that these effectors have a larger potential to interfere with the host interactome, which could explain the selective pressure to maintain them in most strains. Since the SLiM encoded by PpPRxLR1 gene is a potential ubiquitin-related domain, it was interesting to show that PpRxLR1 could potentially interact with plant U-box type E3 ubiquitin ligases (PUBs) to form a network with 18 other plant proteins.

To validate our predictions on the PpRxLR1 interaction network, we ran AraPathogen prediction analysis with a previously characterized RxLR effector, PexRD54 (63). This effector was chosen because its structure has been experimentally solved and also because it has been shown to interact with autophagy-related protein 8 (ATG8) (63). A total of 145 potential interactors of PexRD54 effector protein were recorded (Table S4). Similar to PpRxLR1 interactors, the majority of PexRD54 potential interactors were shown to be transcriptional factors. It was particularly interesting to note that among the 145 potential interactors of PexRD54, ATG8 recorded a significant hit of E-6. This finding suggests that this predictor generates a list of potential interactors of an effector protein, where some could be specific interactors while others are nonspecific interactors. This is also a typical feature depicted by some *in planta* methods, such as coimmunoprecipitation-mass spectrum, used to screen for potential interactors of effectors (64, 65). Therefore, experimental validation of these interactors using effector protein-specific methods like pairwise yeast two-hybrid and bimolecular fluorescence complementation (BiFC) is recommended.

To determine the function of the predicted interactors of PpRxLR1 effector, GO analysis of these proteins in the network was carried out. We found 72 “biological process” ontologies, 10 “cellular component” ontologies, and 11 “molecular function” ontologies shaping the interaction profile of PpRxLR1 (Fig. 6B to D). Most of the predicted proteins were preferentially involved in specific biological processes, such as transcriptional regulation, biological regulation, and gene expression, as well as metabolic processes (Fig. 6B). In terms of cellular components, the majority of these proteins were associated with membrane-bound organelles, specifically the nucleus (Fig. 6C). Furthermore, molecular function analysis showed that these host proteins are significantly enriched in DNA-binding activity (Fig. 6D).

### Structural modeling of PpRxLR1 effector protein.

**FIG 7:**
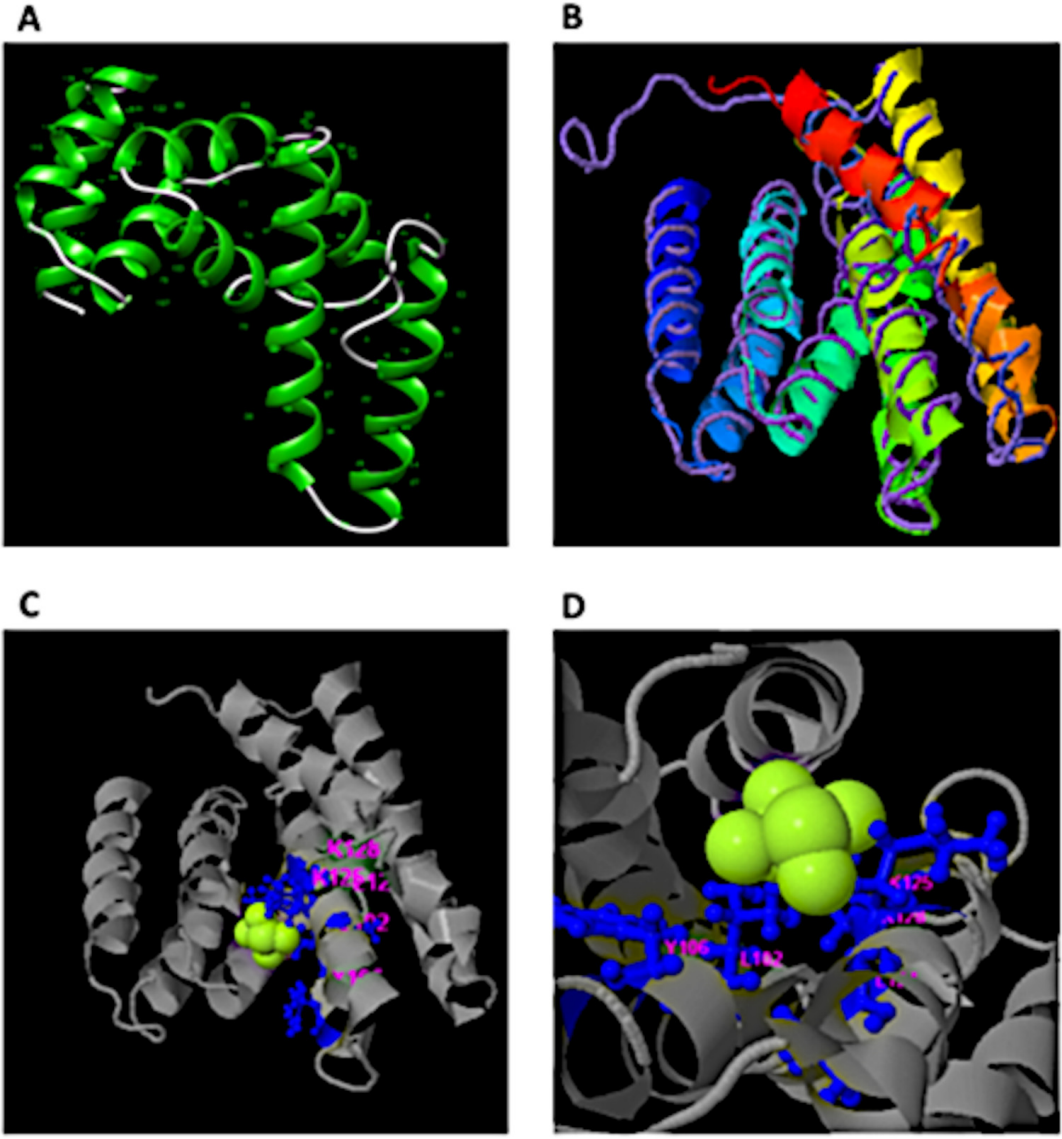
I-TASSER-modeled structure of the complete PpRxLR1 protein sequence (residues 1 to 199) and potential ligand-binding sites predicted by COACH. A ribbon-style representation of the predicted 3D model (PpRxLR1 model 1) is shown, with a TM of 0.54 ± 0.15 and a C-score of −2.19. The predicted structure depicts a modular repeat protein with rigid helical junctions. (B) TM-align structural superposition of PpRxLR1 model 1 (colored ribbon) and experimentally solved 6W2V (backbone trace) with a TM score of 0.951. (C) PpRxLR structure in contact with ligand di-(thiomethyl)-amine (DTN) from lateral view. PpRxLR1 is shown in ribbon (gray) and ligand in the space filling model (green). Potential ligand-binding residues are shown in pink. (D) Close-up view of these residues, including L^102^ and Y^106^, are directly involved in ligand-binding site.

Among the many predicted interacting partners of PpRxLR1, proteins associated with ubiquitin-proteasome system were recorded. Interestingly, the SLiM carried in this effector was predicted to mimic host ubiquitin-associated domains. We therefore reasoned that the SLiM in PpRxLR1 could promote ubiquitination of the host immune regulator, hence affecting the 26S proteolysis system. For us to gain an insight into this hypothesis, structural characterization of PpRxLR1 is crucial. An insight into the three-dimensional structure (3D) of a protein is a key component in determining the impact of a mutation in causing disease. Iterative threading assembly refinement (I-TASSER)-based local meta-threading server (LOMETS) was used to model the PpRxLR1 3D structure. The first of five models obtained as a result of a full-length simulation from the PpRxLR1 sequence was obtained as a 3D coordinate file from the I-TASSER online server (Fig. 7A). The predicted PpRxLR1 model 1 had fold topology similar to that of 6W2V (66) (Fig. 7B).

We employed COACH server to search for ligand as well as their respective binding site for the proposed model based on I-TASSER prediction. Five putative ligand-binding residues, L^102^, Y^106^, L^124^, K^125^, and K^128^, were predicted (Fig. 7C and D). It was interesting to note that residues L^102^ and Y^106^ corresponded to the SLiM motif identified in PpRxLR1 effector. In addition, sequence alignment (Fig. 5B) shows that residues Y^106^ and L^102^ are highly conserved among PpRxLR1 orthologs, insinuating their crucial role in ligand-receptor binding.

We further validated the predicted structure of PpRxLR1 full sequence predicted using algorithms MolProbity and ProSA web. Both algorithms recorded that the modeled PpRxLR1 structure was of good quality (Fig. S4).

To further substantiate that residues L^102^ and Y^106^ are important for the biological function of PpRxLR1 effector, we substituted these residues *in silico* to alanine and assessed its impact on protein function, structure, and stability. To increase the confidence in prediction of deleterious mutations, we have incorporated two algorithms (Provean and Polyphen-2) for protein function and two (Mutpro and I-Mutant) for structural function. As expected, mutation of the two residues predicted significant negative effects on both the function and structure (Table 1). Since L^102^ and Y^106^ residues are evolutionary conserved (Fig. 5B), it is therefore likely that their function in PpRxLR1 is crucial.

**TABLE 1:** *In silico* analysis of point mutations of L^102^ and Y^106^ and their predicted potential effect on PpRxLR1 structure and function

Tool	Score		Comment
	L102A	Y106A	
Function			
Protein variation effect analyzer, (PROVEAN)	−3.167^*a*^	−6.667^*a*^	Prediction (cutoff = −2.5)
PolyPhen2.0	0.816^*b*^	0.816^*b*^	0–1; a higher score with high sensitivity and specificity indicates the higher damaging effects of missense mutation
Structure			
Mutpro	−2.35^*c*^	−1.26^*c*^	G value of ≤−1
I-Mutant	−1.05^*c*^	−2.23^*c*^	ΔΔG < 0

a Deletirious.

b Possibly damaging.

c Decrease stability.

### *In silico* molecular docking and dynamic simulations.

After establishing the reliability of the AraPathogen predictor, we conducted an additional validation analysis to confirm whether PpRxLR1 could interact with some of the predicted interactors. To this end, we chose two ubiquitin-associated proteins (autophagy-associated protein [Atg8] and ubiquitin activating enzyme subunit [UBA3]). These two proteins were chosen for the following reasons. (i) The predicted SLiM carried in PpRxLR1 effector was shown to be a potential ubiquitin-associated motif (UBA). (ii) Among ATGs, ubiquitin-like protein ATG8 plays a central role in autophagy. The ATG-interacting proteins usually contain a ubiquitin-interacting motif (UIM) for ATG8 binding. (iii) Several ubiquitin- and autophagy-associated proteins are highly conserved in plants and expressed during pathogen invasion, and most importantly, their structures have been resolved experimentally.

**FIG 8:**
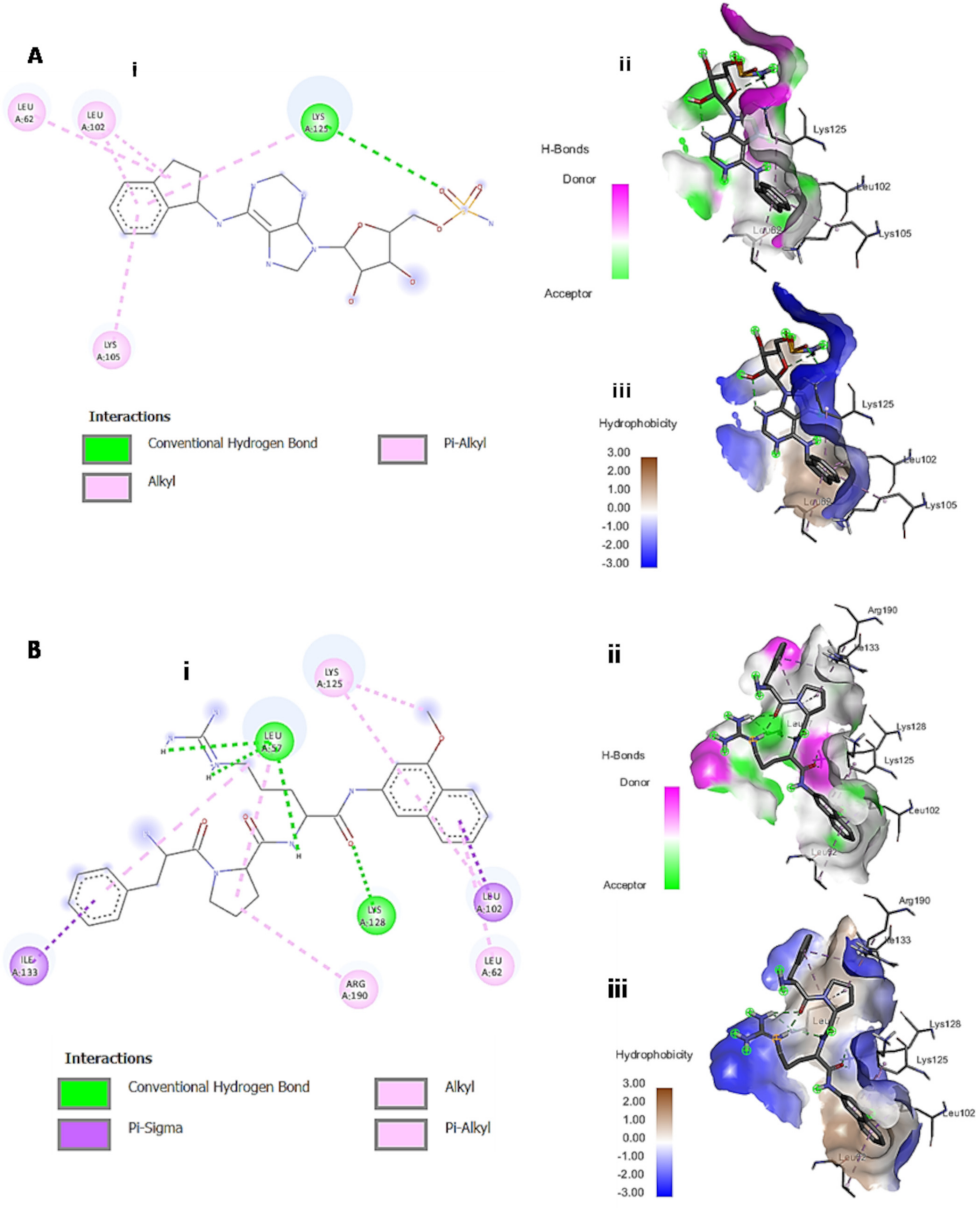
Docking interactions of PpRxLR1-UBA (A) and ATG8 complex (B). (i) 2D docked view of complex. (ii) Hydrogen bond interactions of complex. For H-bond surfaces, H-bond donors are colored as magenta surfaces while H-bond acceptors are colored in green. (iii) Hydrophobicity surfaces of complex. The hydrophobicity of the amino acid residue surfaces is colored from blue for hydrophilic to brown for hydrophobic, accordingly.

Molecular docking analyses were conducted to hunt for the best binding modes or the conformation of the ligand with the active residues of PpRxLR1. The binding affinity scores of the ligand-PpRxLR1 modes ranged from −7.6 to −7.1 kcal/mol and −7.8 to −6.9 kcal/mol for UBA and ATG8, respectively (Table S5). These high binding energies affirm the predicted interaction between PpRxLR1 and the ubiquitin-associated proteins. Molecular docking results further revealed that PpRxLR1 interacts with UBA and ATG ligands through the formation of a conventional H-bond and alkyl and pi-alkyl bonds with key residues of PpRxLR1 (including Leu^102^ and Lys^105^) (Fig. 8, i panels). This implies that PpRxLR1-encoded SLiM could be mediating the interaction between the effector and UBA/ATG8 molecules to perturb host immunity for disease development. To determine the stability of our docking results, the molecular dynamic simulations (MDS) analysis of root-mean square deviation (RMSD) indicates that the protein–ligand complexes obtained a stable conformation during the simulations with a few conformational transitions (Fig. S5).

## DISCUSSION

In this study, we adopted a conventional *in silico* pipeline to identify hundreds (165 to 358) of RxLR effector genes (67, 68) from the genomes of 11 strains of the wide-host-range pathogen *P. parasitica*. This high number of effector proteins is commonly attributed to the complex relationship between *Phytophthora* spp. with their respective hosts that enabled these effectors to stay a step ahead in the evolution of plant resistance gene (*R*) products and effector recognition (46).

Despite the high number of these RxLR effectors, a subset of these were found to be conserved among all the 11 strains. Here, we predicted that *P. parasitica* strain INRA 310 may secrete a total of 333 RxLR effectors, out of which 71 are conserved in all *P. parasitica* strains. These 71 core effectors probably have a more generalized and host-independent function, such as suppressing plant immunity (49, 69, 70). Since core effectors have the potential to target conserved plant proteins and processes (17, 71), they can be harnessed in breeding for durable as well as broad-spectrum resistance in plants (43, 72).

Generally, for every gene, the distances to its closest gene neighbors in either direction are designated its flanking intergenic regions (FIRs). FIRs can be employed to ascertain whether a gene resides in a gene-dense/core or gene-sparse environment (73). In this study, we show that RxLR effector genes are found largely in the gene-sparse regions of the *P. parasitica* genome. Studies have shown that genes that localize in the repeat-rich and gene-sparse regions of the genome are likely to evolve faster than core genes localized in repeat-poor or gene-rich regions (12, 51, 74). In fact, it has been proposed that *Phytophthora* species have what are termed “two-speed genomes,” where effector-coding genes reside in gene-poor, repeat-rich regions that are prone to rapid evolution and responsible for host adaptability (12, 51, 75).

It is worth noting that the essentiality of these CREs is not likely to be retained through specific protein domains. Existing studies have revealed that functional domains could be found in the unstructured regions of effector proteins commonly known as intrinsically disordered region (IDRs) (76, 77). There is increasing evidence to support the occurrence of IDRs in effectors, particularly in bacterial effectors (77–79).

Similar to bacterial effectors (80, 81), we showed that CREs of *P. parasitica* carry IDRs with N-terminal regions (RxLR-dEER) highly populated by disordered residues, while the disordered content was depleted in the C-terminal regions. We speculate that the disordered state of the RxLR-dEER region may provide an inherent advantage and contribute to RxLR effector translocation since this region is implicated in effector delivery into the host cell (67, 82, 83). Bacterial effector delivery into the host cell via type III secretion system and the translocation of disordered regions would be of native advantage, as it would spare the active unfolding, which is required prior to delivery (77, 84). Reduced IDRs at the C-terminal regions of CREs could be attributed to the presence of WYL motifs (41).

The C-terminal region of RxLR effector proteins is central for the effector activity. In this study, IDRs were also recorded in this region. This was expected since IDRs of virulence factors have been shown to carry mainly functional domains/motifs molecular recognition feature (MoRF) and short linear motifs (SLiMs), which are responsible for interactions with globular protein domains and mediate a wide range of important cellular tasks (85). Here, the majority of SLiMs in CREs belong to the ligand (LIG) class, while the rest are grouped as targeting (TRG), docking (DOC), modification (MOD), or cleavage (CLV) motifs. This significant variation in classification could be attributed to the fact that RxLR effectors are secreted into the host cell purposely to interact with various immunity proteins, thus crippling the immune response (16). In addition, some CREs were predicted to have more than one SLiM, which is hypothesized to provide important functional benefits for interactions with multiple partners. The ability of RxLR effectors to interact with multiple protein partners is perceived as a strategy since they could interfere with plant protein interaction networks (86). Coupled with an extensive review by Mondino et al. (87), we propose that SLiMs encoded in CREs may interact with plant proteins to mimic plant immune signaling components and manipulate plant responses for disease development. To this end, we focused on *P. parasitica* effector PPTG_01962 (PpRxLR1), one of the CREs that is expressed specifically in the late biotrophic phase of infection (30 h) in *P. parasitica* (59).

Protein-protein interaction (PPI) networks can highlight the modularity of cellular processes and allow the deciphering of protein functions at the cellular level, as proteins tend to interact with each other when they are involved in the same molecular complex, pathway, or biological process (88). We generated PpRxLR1-host protein interaction network using IntAct tool on Cytoscape. Interestingly, PpRxLR effector was predicted to interact with 161 *A. thaliana* proteins, forming a fuzzy interaction network. Previous studies have shown that specialized pathogens employ effectors to manipulate the key components of their hosts’ intracellular networks to their advantage (89, 90).

Protein stability is crucial to proteins’ activity, function, and biomolecule regulation. Any incorrect folding and/or decreased stability are the main consequences of deleterious missense variants. In this study, we show that *in silico* analysis of the functional and structural consequences of Leu^102^ and Tyr^106^ mutations suggests that the mutations could interfere with function of PpRxLR1. This can be used in identification of potential targets of this effector.

Molecular docking is crucial to screen larger data sets of small molecular to achieve potential lead-like molecules for a specific target. In this study, molecular docking output revealed good binding affinity values ranging from − 6.9 to −7.8 kcal/mol, which confirms the potential of PpRxLR1 to interact with ubiquitin-associated proteins. In addition, molecular dynamics simulation (MDS) is an important method to assess the stability of a protein–ligand complex in dynamic states. Based on root-mean square deviation (RMSD) analysis, we show that the interaction between PpRxLR1 and its ligands (UBA and ATG8) is stable. Hence, both molecular docking and MDS might be crucial to validate potential plant proteins that are targeted by phytopathogen effectors. Existing studies reveal that *Phytophthora* spp. secrete RxLR effectors to modulate the host’s ubiquitination system as well as autophagy process for their own benefit (63, 91, 92).

In summary, *P. parasitica* secretes a plethora of RxLRs, with a subset of these being conserved (CREs). Intrinsic disorder is a common structural characteristic of these CREs with SLiMs carried along these disordered regions. Due to their small size and structural flexibility, SLiMs provide functional advantages for effectors to mimic host regulatory units causing the pathogen to trick and sneak into the host territory for disease development. Although these *in silico* analyses offer a theoretical platform for the molecular mechanism of PpRxLR1 during *P. parasitica*-host interaction, *in planta* analyses are recommended to authenticate these findings. In addition, this study identified a total of 71 CREs in *P. parasitica.* Focusing on CREs could be crucial in engineering plants with broad-spectrum resistance; however, it is highly recommended that the identified CREs should be screened for the presence/absence of sequence polymorphism.

## MATERIALS AND METHODS

### Data sets and identification of RxLR effectors.

The protein sequences of *Phytophthora parasitica* were retrieved from the National Center for Biotechnology Information (NCBI). For RXLR effector identification, the presence of a signal peptide (SP) was predicted using SignalP v5 with the criteria that cleavage sites had to be located between residues 10 and 40 and hidden Markov model (HMM) probability of ≥0.9 (66). Transmembrane domains (TMs) were predicted using TMHMM v2.0 (93), while the presence of RxLR motif was scanned using HMMscan (67). Putative RxLR sequences were further analyzed for orthology using eggNOG v5.0-mapper v2 (94). The amino acid sequence of PpRxLR1 was used for sequence alignment with homologues from selected *Phytophthora* species using Clustal Omega (https://www.ebi.ac.uk/Tools/msa/clustalo/) and BoxShade (http://arete.ibb.waw.pl/PL/html/boxshade.html). Sequence conservation profile was obtained using the WebLogo server (http://weblogo.berkeley.edu).

### Genomic architecture analysis.

A detailed method by Saunders et al. (73) was employed to analyze intergenic distances. Briefly, the 5′ and 3′ intergenic distances for all genes as well as the identified RxLRs (conserved and nonconserved) were two-dimensionally binned in R, and a heatmap graph was plotted using ggplot2 R packages to facilitate analysis and visualization of whole-genome architecture. Wilcoxon rank-sum test was carried out to determine the statistical significance of the difference between mean intergenic distances between conserved and nonconserved RxLR effector genes.

### Prediction of intrinsic disorder.

The level of intrinsic disorder for RxLR sequences was calculated using PONDR VL-XT, disordered predictor (95). This predictor has been shown to be more accurate than other predictors since it integrates three different neural networks and was trained using experimentally confirmed disordered protein regions (95, 96). The predictor uses protein sequence as the input and calculates the sequence average disorder at each amino acid position in each RxLR sequence and aligned on the first amino acid. We computed predicted percentage of disorder, which represents the mean disordered residue content for each RxLR protein.

### Prediction and classification of SLiMs.

Short linear motifs (SLiMs) were predicted by ANCHOR tool (56). A peptide is classified as a SLiM only if (i) it resides in disordered region, (ii) it cannot form enough favorable intrachain interactions to fold on their own, and (iii) it is likely to gain stabilizing energy by interacting with a globular protein partner (56, 57). We further classified the predicted SLiMs using the eukaryotic linear motif (ELM) database (58). The database annotates SLiMs into six classes: cleavage sites (CLV), degradation sites (DEG), docking sites (DOC), ligand-binding sites (LIG), posttranslational modification sites (MOD), and motifs for recognition and targeting to subcellular compartments (TRG) (58).

### Amino acid compositional profiling.

Disordered regions are characterized by their compositional bias toward polar and charged residues. This means that IDRs encode a high content of disorder-promoting residues (Ala, Glu, Lys, Arg, Gln, Ser, Gly, and Pro) and a low content of order-promoting residues (Asn, Cys, Try, Phe, Tyr, Val, Leu, and Ile). On the other hand, amino acids Asp, His, Met, and Thr are not consistently enriched or depleted among intrinsically disordered proteins, so they are considered disorder-order neutral residues (97). Amino acid compositional analysis was performed by Composition Profiler (www.cprofiler.org) using the PDB Select 25 as reference for ordered proteins. Enrichment or depletion for each amino acid type was calculated as (*C*_x_ − *C*_order_)/*C*_order_, where *C*_x_ was the absolute composition of each amino acid in the RxLR data set and *C*_order_ was the corresponding value for the control set of ordered proteins from PDB Select 25.

### Protein-protein interaction network.

The PpRxLR1 protein was submitted to the AraPathogen predictor that predicts protein-protein interactions (PPIs) between Arabidopsis thaliana and pathogens based on sequence and Arabidopsis thaliana intraspecies PPI network (InterSPPI) (62). This predictor was found to be suitable because its training set consists of pathogen effectors and their host targets. The cutoff significant interaction for this predictor is E−05. Any potential protein that scored an E value that is greater or equal to E−05 was not considered. The interactions were analyzed at their highest confidence level using Cytoscape (98). The popularity of Cytoscape tool is attributed to its open-source, modular design, which affords great flexibility and extensibility (99).

### Functional annotation of potential interactors of PpRxLR1.

Functional enrichment of the predicted interactors of PpRxLR1 was analyzed using two approaches. First, the collection of enriched GO terms resulted from database for annotation, visualization, and integrated discovery (DAVID) analysis. Second, the obtained terms were then summarized and visualized using REVIGO web server (http://revigo.irb.hr) (100). This analysis assists in reducing the number of redundant enriched GO terms using a simple clustering algorithm and produces a scatterplot, relying on semantic similarities. For this analysis, the enriched GO terms and the *P* values from DAVID were uploaded to REVIGO. Settings used for REVIGO program were as follows: database, Bos taurus; semantic similarity, 0.7 (medium); semantic similarity measure, SimRel.

### Structural modeling and classification of PpRxLR1.

Physicochemical properties of PpRxLR1 protein were analyzed by ExPASyProtParam (https://www.expasy.org) server, while its secondary structure was characterized by online tool https://www.compbio.dundee.ac.uk/jpred/. In the absence of experimentally determined PpRxLR effector protein 3D structures, homology modeling was employed since evolutionary-related proteins are believed to share a similar structure. However, in our study, homology modeling was not suitable since the percent similarity between the target (PpRxLR1) and template was <20%. Therefore, the 3D structure of PpRxLR1 protein was developed (http://zhanglab.ccmb.med.umich.edu/I-TASSER/) (101).

### Ligand-binding residues.

The prediction of ligand-binding site and possible ligand-binding residues of PpRxLR1 was generated using COACH protein-ligand-binding prediction server, a meta-server approach to protein-ligand binding site prediction (http://zhanglab.ccmb.med.umich.edu/COACH/). The complementary ligand-binding site was predicted using COACH by matching the PpRxLR1 I-TASSER generated model with protein in the BioLiP protein function database. Also, the functional templates are detected and ranked by COACH using composite scoring function, which is based on structure and sequence profile comparisons.

### Structure model validation.

Structural validation quality of the predicted structure was analyzed using structural validation algorithms ProSA (102) and MolProbity (103). ProSA compares the Z-scores of the predicted structures against protein structures of the same size obtained by nuclear magnetic resonance (NMR) and X-ray crystallography. MolProbity is an algorithm that validates the general stereochemical quality of a protein.

### Point mutation on PpRxLR1 function and structure.

To determine the effect of mutation on the function of PpRxLR1, both PROVEAN (protein variation effect analyzer) and PolyPhen 2 were employed. PROVEAN is a software tool that predicts whether an amino acid substitution or indel has an impact on the biological function of a protein where a threshold of −2.5 was used (a score of ≤−2.5 was considered “deleterious” while a score of >−2.5 was considered “neutral”) (104). PolyPhen 2 utilizes straightforward physical and evolutionary comparative considerations to predict amino acid substitutions on protein structure and function. PolyPhen 2 calculates and computes the difference in the position-specific independent count PSIC score of the two variants (http://genetics.bwh.harvard.edu/pph2/). The probabilistic score ranges from 0 (neutral) to 1 (deleterious), and functional significance is categorized into benign (0.00 to 0.14), possibly damaging (0.15 to 0.84), and probably damaging (0.85 to 1).

The stability of PpRxLR1 upon single amino acid residue mutations was predicted using Mupro (http://mupro.proteomics.ics.uci.edu/) (105) and I-Mutant 3.0 (http://gpcr2.biocomp.unibo.it/cgi/predictors/I-Mutant3.0/I-Mutant3.0.cgi) (106) using default settings. Mupro and I-Mutant 3.0 are valuable tools for protein stability prediction and analysis, even when the protein structure is not yet known with atomic resolution. Both use support vector machine (SVM)-based tools to predict protein stability changes for single amino acid mutations from both sequence and structural information, which correctly predict protein stability changes with over 80% accuracy using cross-validation methods (data sets and experimental) (105, 106). PpRxLR1 protein sequence was searched against the web server, and energy changes (ΔΔG) were recorded. A negative value for ΔΔG represents a decrease in protein stability, whereas a positive value for ΔΔG represents an increase in stability.

### Molecular docking analysis.

The homology-modeled PpRxLR1 effector protein (PDB format) was downloaded from I-TASSER (101). The modeled protein was considered the receptor, and the complexed ligands were manually removed using Discovery Studio. Furthermore, the docking simulation of two optimized molecules (UBA and ATG8) was done using the PyRx virtual screening software (AutoDock Vina). In addition, the Vina wizard employed a gradient algorithm search for predicting the binding scores and modes of the ligands in the active sites of the receptors. The docking result with the highest binding score was visualized to assess the molecular interactions with the aid of the Discovery Studio Visualizer v16.1.0.15350.

### Molecular dynamics simulations.

Molecular dynamics simulations were performed for PpRxLR1-UBA/ATGs complexes. The docked structures of the effector protein with the potential ligands were taken as a starting point for simulations. Simulations were conducted in a periodic water box for 40 ns using the CHARMM36 force field the and NAMD package version 2.13. The force field for ligands was generated from the CHARMM-GUI server. The water box (including 150 mM NaCl) was created by adding water for 20 Å in the positive and negative *x*, *y*, and *z* directions around the protein, yielding a cuboidal box. L J cutoff was defined at 12 Å, with a switching distance of 10 Å. Long-range electrostatic interactions were handled using the particle mesh Ewald (PME) method. Before the production run, the systems were minimized for 5,000 steps using a conjugate gradient algorithm. The simulations were performed in an NPT ensemble; the temperature and the pressure of the system were fixed at 303 K and 1 bar, respectively, using a Langevin thermostat and barostat. Postsimulation analyses were performed using VMD (root-mean square deviation [RMSD]).
