# Supplementary material for: Short Linear Motifs (SLiMs) in “Core” RxLR Effectors of Phytophthora parasitica var. nicotianae: a Case of PpRxLR1 Effector
Source: Microbiol Spectr. 2022 Apr 11;10(2):e01774-21. doi: 10.1128/spectrum.01774-21 (PMC9045269; doi:10.1128/spectrum.01774-21)
Supplement: SUPPLEMENTAL FILE 1 — Supplemental material. Download Supplemental_File_1.pdf, PDF file, 0.11 MB [file supplemental_file_1.pdf]

Table 1: List of 71 core RxLR effectors in P. parasitica inra 310 strain

>XP_008916175.1
MRWYHVLLILVSSFQTISNAFSTSPITTVASPAHSISTSFAVTETARRRLLRVVNRANAESNSFKDVGYGENTGDVSGYSGYDKSS
IKDLEERVTGTEAFQKLKSSKTFKSFSQGVTKATNTFSVKISPALPMKAKLQVWSNNGKSVSFVRKELGMDKLDDALLKQAKNF
QFYDDYVTSQLPIWAKNELTLDDIVSQLGLRGLSGSDLTSNPNFKYYDQYLEHQALVWATKDLDLDDVLVRLDLNILPAAERLG
AVNFKFYEEFVAGLMRSWMEKGTPLDDVMAKLKLDKLTGEELLKHPNYRYYKNHVKNYLRVWAFNGESLDDVAVWLGLEN
MQGKLLESQPNYAFLKKYWDKSTKYEEQEMLKRRITSFQVWDDLQVFRVKQSRRKNSETYKAYKHYVNLIDDYIIRLKDRGFTE
DELPRMTSKNAAPDEMMEKTYIWTQMRRPEWYVKFSLGLDGLGENALKEAANFQYYEYYLRAMKAVNPTVQ

>XP_008913097.1
MRLSYLLVVAATGLLASANAATESTETTMTKTAVDFVNGKRFLRVSSTDDYDNGNNDEPKSKKETSTTTNDDANSKKDEERGII
VDGIYQDDYQRWFKEGKTPAELADDLGLDEFSILGHPLERRIYRGYKRFYRVACEDGECEKTKRGRSDDEEDEDEDE

>XP_008910631.1
MNVSEVVAALLLSVLIFPTVIIAGKTTDNGPTANFLRSDPYKLQDYEERGKMPRLADVGEKINSIVKSTQQKIWLTYGRPAEDLLS
RMTSVLKEDNPLANSKLQVWLKHVDDLNQKHPREAKSAASMLTAHYGDNQYGNSLLSTKLNAAMENEHTKVFALKLQAQRL
EDWGRKNTAPLAVFDMLLLRNHDVLKNPLLISWFKYVEDFNARNPVDKMTLISTLSLGFGTSDRGLVEVLEAGLNAGGTKDIA
TKLKTQLFAEWEAKKLTPDLVFDSTLRLKQGYTDSNAPGLSDPILKFWIRYMEWFNLKHPHKQKTTLFDTLRNHFDYDAIVRML
VDAKLDPASKASARDLESLLFAKWLDGKLTPQDIARWLRADRSDAMFDEYKRLFEMKWPNGV

>XP_008917288.1
MPNPTMWLQSTALLYAVVTLLLYTESVSASAISGRASADLPTTHRMQLDKPQRIYTRRLLRGTLANDERNENRVINGAIEKLAG
LAKTGALKISENARYWLGREQPADEILR

>XP_008910741.1
MRCRWFILLITVTLACNAQAESKTKIHSITPAEIHSNPRVLRAYKTTNNIDEGFHDEERVASGSAVETVTNSLKSTSTQFDDWLAK
GTSTDDVFKLLTLDKPSEGLLTNSKLTEWIHYMKLFNKANPKKQTSLIKTLTAHYGDEALVKMIDAAKQVPSTATFAKRLQTEQR
QLWLTQGKSPDDVFALLKLNKAGDKIFTHPEVVTWANYVGDFNKANPDNPVSLFSTLATRFNDETLVPMLIAAKKNPSTEKIA
VQVQAVQTKLWLKDEKRPEYVFNLLRLNREGRDIFKSPLFTAWVQYTDDFRKIYYGTDFTTIATLTKYYGDENLTRMILEALNSP
STANIAKRLEIEQLRNWYVQKFTPKDVFNALNLYSEGVKSFESPLYTVWTKYATYLSAAEPEHKRKQPSKSSPSG

>XP_008911091.1
MPRRCVVLVAAATLLATSTVFSLSTDTTATTHSFAQPHNFAAQRFLRTYTPYSNEERGIDFKSILGVDLKNVWTQQKLSHYLKKG
KNGDDVFEKLKLQNVKAGKTIFENPKFLAWEQYVAKYNANPLNEEISMIATLSKHFDDDVLTKMIDAASKSSVAETKRIGATLQ
EQQILFWRSKKLAPDRVLKQLKLANDVDDLLTSPTFLTLSRYLDDYNAQNKMSLSMTEVLRRGFDEDDVAKMLQQAVLNAKD
AKTKDLAVKLQNQQFDIWKKLGWNGDEIFTKLGLNERGVTLENPNFAAWAKYVEKTTGNEKLPFNTLWKRYGEQTLATMLIA
DRKKLGGNDFVTSMQSHLIEKWLTMIRDPDDVAKILGTSFQGKILTASYRWNFKSAFG

>XP_008911266.1
MGLSNVVLIVAAAILLGSISCTAKNDTPRLVPEIAPYEREYAPTTAGNTNREEKQILRFLEVARVAEGDEEERALNFAYLKNLKNLE
KYIPKADTLKAAMKAAQAKKAAKAADKLKKNNLFKLPGTTDDQLISKFREWRAQGKSSADVTNGIFKTNGMKQNDAITIGNRY
QVYLNDFG

>XP_008901208.1
MRVSYVFLVAATTLLATCDVMAASPSALSKTSVDAVNGHVDISTRSLRMLKKKTAAVDPDDEERVAEARQMDSEILEKIVNDH
AYAKQVYRSWLQNGQTKEDIENRLKTLGLLTKYGNVVKQYGQYLTNLEERSV

>XP_008910670.1
MSSIYWIMWLYSLLGTLATLLVCVESASISATSDPDTFQELSLKPEGAATDRLLRSADTSDELSENRVTNAGIEKITNLFKTGVTKT
KTSGQHSADEILKKFKLENGIEEALVSPNLKAMEDYVKDLNSKNRFKKTSMIRLFTKYYGDDVVAKALSKVQGSAHSADAVDTIK
QLRMEQLTAWLKSKKSVDDVFKLLKLRDDSLMAFVSPKMAMLDDYIKLFNREKSSGKTLAKTLVTGFGESQFVSLLQLGLKNSY
TKDLATSLETELVNLWLASKFKPGDIMKKLKLDEDMGLALANQSRQSRELLTRYISVYNQRYPNSKTSLVATFTARYGDDVVAET
LYMARQAPDSKRLAIDLQKQQYMEWQNSGISADDVFKALKVKVEDFATIISPKLESLYEYIKILKTNDLRGAPDDFTVVRSGLGG
DGDLARMLVRSFTVSDALNMDAASSMATVYEKKLFKRWTNDSIEPKTIYSKFLNVEETSANTWEKAFASRYASYFEKKKFSSNL
VNFNDPRRL

>XP_008894932.1
MRSFLLLLVLTLVAITSSNAFMPSRPQLKVSKPELEQATPWRTDDKRFLRASGEVDEERGFSDLAAKLKAWLQKFKSWFTKSKP
VTAATTKYEKVAEKVAIKYQSLMYKSEVAKAEDLIKKGVMNDDLYKNKISPEAYFDAMKLDPKLKFISDSAVARANNPNLEKFFT
YSLFWTKKNEVTKAEGLIKKGVMNDVLYQNKISPETYFDALKLDPKLRFVSDSAVARANNPNLEKFLSYTSFYNKSQAGKREVAK
AEDLIQKGVTDKVLLKNQISPEAYFEALKLDPKLKLIADSAVARKNNPDLEKFYTYATKYYNSLAGK

>XP_008908346.1
MRVHYIVVLLGALATSVSADVSRTDSKIAAPRYARSLPTDRTVVRGLRSNKPRHDSEERGIFDGALAYVDDFVKNLLKERTGWD
LIDDLTENVNLSPKISEMLKKNTPVDEAFKALEVEKVAGIDNLLESQQWKEWAVFVIKRSKDTPDEALSSAMSIQFGLDRVSSILA
NAGKNPKTKAIAEKLEEAQLKRWLSLGSDPDKIFEALKLNTKFDSFFDSPQFATWTSFLKAFNAKNSNKKEITGLDVLLQYYKEDS
LTKLIVSADNVNSPRAQQYLDELLLRWRDQPLHPQHVFTMMKLDEAGDALLTNPLLSRWVKYMEDFNEKYPLAKTSMIQTFT

KNYGDEKVATMLQAAAKASDADTAKIASTLQEAQFKNWMVLGLTPDEVYNTVLKITSTSSPKADIWRAYYNAYEKEFPGKLFS
FNP
>XP_008910627.1
MRCHYVVLLLAVNIVASIDANSMTTETASLGPSHTVQDNGVSTIRYLRSVATGEEDEERVVVMPGLNSIKKNPVLQNVNKVSN
RAKSIITSATLLKWAKQQRSPDLVFKKLKLDKTGSKLFESPAFNMWVTYTNKIFKTDADSVIFTSLSKRYGVAGLMRMTETATKV
DSTKGMATKLQNIQLDDWKKLGMSSDDAFKALKLNEKVDDLLTNPNLNTWVKYVELTKKNTRLNTPMIDTFRAHFKDDALLK
MFKAAQSNPKTKRMGVHLENSLTNIYRLQHLKNTS
>XP_008894926.1
MRSFLLLLVLVFVAITSSNALLTASHRHQPKTAKLKLDQAVQWNIEDKRFLRATDAADEERGLAGIKTKLKAWLEKFTSLFKKSKS
AKAAATTTTTNLEEVAEKVAIRYQSEMYRSEVTLAQDLVKKGVVDDVLYQNKISPEAYFDALKLDPKLKFISDSAIARGNNPNLEK
FFTYSLFWTKKNEVTKAENFIKKGAVNDVLYQNKISPEAYFDALKLNPKLRFYSDSAVTRVNNPNLEKFLSYTSFYNKSQAGKREV
AKAEDLIQKGVTDQVLLYNKISPDAYFEALKLNPNLKFIADSAVARKNNPDLEKFYTYATKYYNSLTGK
>XP_008895051.1
MRSFLLLLVLALVAITCSNAFMSSRPKLKVSKPELDQAVQWNIEDKRFLRATDAADEERGLADTKTKLKAWLEKFISLFKKSKSAK
ATTTTTTTNLEEVAEKVAIRYQSEMYKSEVMLAEDLIKKGVMNDDLYKNKISPEAYFDAMKLDPKLRFVSDSAVARANNPNLEK
FFTYSLFWTKKNEVTKAEGLIKKGVMNDVLYQNKISPETYFDALKLDPKLRFVSDSTARANNPNLEKFLSYTSFYNKSQAGKREV
AKAED
>XP_008893512.1
MHLPHVLLLALCIAIASCDAISAFVTEQEAAVVQNAQRLGLSHSVVAARDRGASGKRLLRSDNLPTVTNDVDAEERALPGITKLS
ELAKKGKSAVSTKLSDKMLWLKYQKLGKQKLSDLDITGMWLKSGKGPDKIFDRWIRLSKSPKQAAQNLLNHGTTTNDLYKVLR
KRNMNLETIRPIWRDLGLTENQLRVARHAVSAL
>XP_008916752.1
MRLAYILVVLIAGTLHANSNAFPVVADAKTMIKNGGSPDIFASTHTDDHRLLRRVENYEGVVEEEGGGFKDKLKAVAEKLNPVK
AAEKAKDKAKEIKDKVLESDWDKLVKHLQIHGDNKG
>XP_008903800.1
MRLHPALLAVALACLASCTQTLATSVESSSTKWSPVITSNTFYRARLLRGVATSDDAEERDFFSQMAEAVAKWGTTTALLSLGK
TDDEAKKILGLEKLSGEALKSHANYHILDEFLSKLRSRKVTQWLNKDTTTDEVWKALQLDDLSTKLDAKEFKQSEALKTYVEYVKK
LDDDIVKFKRASFEPDNSSPLELAVKIHIWAKAGRDPAHALEIMGMNALKGSKNRKFYEEFLDLINGRKPIIDY
>XP_008906679.1
MRGRHVLAFVVLAAVCSSRCEAVGASSERLKQTKQATSGATQWTPRLVLDEEPKRSLRRGAEVLNLGRDAAFKAQLKLKLQVS
LAVGLKPDQVLILLQPTSTKDTLYRHYTKYFYDYFVKYLDEPMSHLPQQVIKNIWEARLHAWLQTDTPPQVFSKLKLVKSMDTD
TFASAKGQKNYDIFEEFHKRWMRKQQKESTKPKLDIVIHRNDGT
>XP_008894116.1
MGLLYLVGLASLAAITSTDANSPIVTSSVQSPKVSNWSSSTSVVSTTRFLRTSDTDDEESEERAGLSVPASEKLKSWFMSSNVNP
RQLQKWLNEGKSGETVFTRMHLTNVGSLLFYDPQFKTWLQYVDDLNAKTYQKRTPAISVLTTQYGDDALYKMIVDAKMIPRM
KELASKLQADQMDHWVAVAKDPDEVFHLFKLDKLGKTRMKLFSSPEFAAWAKYMDDLSMNNPEKARPMISTLRQHYRDVD
LLTMAESVKSVEATKSIATRLETEVIKDWAVSRKTPDKALRDLDLDNADTLLKDPLFNFWAKYVDVYNARYPEEKMTMIKTLTQ
KFDDNNVAKMINAAKANDATKDIAAKLEMAQLQMWLHDRRSVDDVLVRLWIHTTENDFLGNPLLNTWVAYMNTVITENPT
KVSSIFSVLETRYSDKALLQILEVAKGFPSMKNTATKMQKKKIQAIFARWELPSKAFGLLGLDRIGDNILSTPLFRRWMHYVEVFN
KKNPDRQESWIDPIRFNYGWSGVEGAIKQAMKNPKSVNIAKQAESAWLDTWLDAAKPPEDAFRFLHLDNVFENSLSSPKFAT
WAKYLDDFNKRYSEQKTTMIDGLRANYNDRWLLRIFDAAKSDLNTEKLAANLQNALVDTWLAAKKKPADLKRMLNGVPTSD
QMIERYVKKFDALLENS
>XP_008909301.1
MRFSYFLSLALATLLSSCNANAAISGNNHAKQPSMISSDTAVLSRPIDASNVKRFLRTYRGDHEDKEDKEERRNPALLDEEELVR
WTTKWAARADDWFEQGYTPAQMRDKLTGLDGVMNQKNGRKFYLFVKKWRSEHPNDPGI
>XP_008913692.1
MRIAFVLLFVSAIVLVDSADALFPTAVRSERVLQGAQSGGMRSLRVHNEERGLPFFGGKSSNTKVSTTWLRDTTVYDDILTNAG
FTTAFGIWKQNKYSEGKITRAMNKLGRTQDEIDRVLAGYRKFKNVKVR
>XP_008910577.1
MRLFNMTIVLLAALLLASGTAVSHDDQASVLNVDVVHSSRILSGEDKRFLRSHQTTGDEGKMTEHDNEERLGGDNLFYGLKLS
KMGKDANYRDKVFQRWKNYGHTVDSVTEKRVPDSLIDAFEKYLELRKRTGKVFGDRRPLIEPR
>XP_008891466.1
MRLFNTTFVVLAGAFLASGTTVSKADQTSVANVDVVHSSRVISGEDNRFLRSHQTTGDEGKMTEHDDEERLGGENLFYGLKLT
KMGKDANYRDKVFQRWKNYGHTVDSVTEKRVPDSLIDAFEIYLKLRKKTGKVFGDRRPLVEPR
>XP_008907776.1
MRFTHFLLFATTTLLYSSNASETVSGESQAKLTTVTSTDAAAPARAVDGRNAKRLLRVDREDDENEEDKEERVNPALLDEQALA
RWTAKWAARADDWFDQGYTPGQIKEKLTGLRDEMSRKNGRKYYLFLKKWNSEHPRGRD

>XP_008915605.1
MRVLLLFVVIGYLLSYAAASAAIDAGTTGVVKENSEVGLLSYAIAGGDKQVKRSLRLDNHDELAKLDSKDEERVPSGMVDDVVA
KVGQSKLNDLIKKNLDEFAGFAAQGKKLLGKSSEATKNWGKLKASVLRGKLKPAYEYADQLSLSTLQQLDEIQKLRKVDIKNGVK
GSKKTPDGMRRKFATSNPPKERILPKEEYLVSHVSRDKQRYGKDGSRLLSAGVVTRTNEQGQLQILLISSSNPNKHDFLLPKGG
WDKGENIKKAALREVIEEGGVNAQLAHGLGKVKFKEGEDKYTYFAYLMKSNQIYDDWSESIRYRVWVTPDEAIVMLQNRPH
MQEVVKRAKAMQNKITAGIKPELNPDLAKVKLD
>XP_008893514.1
MRLPYVLLLAVCIIIASCNALSAYEQEAVVAQNAQRWSSTHSVAAAETGDTSGKRLLRSDSIPTVDAEERALPGMTKLTETLKKW
ISALRSKLSNKKLWWNYQKLGKQKLSDLDITGMWLKNGKSYDDIFDRWIRLDKSPRQAAKNLLNHGTTTNDLYKVLRKRNMN
LETIRPIWREVGLTEYQLRAARHAASAL
>XP_008894176.1
MHLTSFLAVSITGTLLTGSNAISDAKAMVENRASSGDIFAPIHSRLLRRAEIDEDEAEEERVLGNMKDKMKAVAEKLNPVKGAE
KMKEKVQETDWSKLVKHLKEQAKREGKR
>XP_008892541.1
MRLTCALLATGIALLACIDCILAATDATKNSQKETPLVAHYSTPDYPKRILRANAQAYSVDGDSEERMFSPMINGVLGKVGSVVE
TGGAKVIKEMPKLEKYQKWLNAGLNPSQVRVDVLKKTPWQALSSILRGKDSYQYLKFIVDYKRLKLGQNIPKPAAQMEKHELW
LYAGLTPSHVRVDIMKKAPDMKLKVIEKHPDFYQYAKFMVEYKRLNPNANIAFVT
>XP_008913100.1
MRFFLVAAVAVIALISSSTDALAETSNAVNSVNDNQGFARSLRSHTTSTEERSVADILSEVDEEDRAVWTVSYRAWYNAKLTPT
QVKTVLGVSQAEMNKVARQLQQLYLGYFSFYTAMEKRKEEKKRLATP
>XP_008895018.1
MPIPSKCMRKVHPGILLATVALLMSIGLALETSNPKTSISTTRLETSGEESNRNQYNRRLRTTTADDDYDSEDRGAPGSAKVEELV
KKSNLQKLGESIKSGFKPDKQEKVTKHFKTLKVDQPEKSKMFSSRLFHEWIRYARKKYKNDWLEADAAMFKSMAEYYGDDVLA
RILADAKHSSGSWIVTRLEKIQLSKWKDIGKDADEVYKILQLDAEGEKLLQSPALGTWVSYVSKLGKNPIDELQVKLSAQKYDDA
VVAKMIALSRYDVNSDFAADLGRSLQKKWKDSDNTESGVFHLLKLDDEKTGLLTNPVLEFWMRYVDSLGKDPYKLLLLAINRK
GINDAGLARMVGLAQKDRNNYWIGTNFESRVLDKWKQDGKSGNKLFTMLELDKEGDKVFDSPVWKIWTSYLNKNEQEPNKI
IYSVEELVKKSNLQKLGESIKSGFKPDKQEKVTKHFKTLKVDQPEKSKMFSSRLFHEWIRYARKKYKNDWLEADAAMFKSMAEY
YGDDVLARILADAKHSSGSWIVTRLEKIQLSKWKDIGKDADEVYKILQLDAEGEKLLQSPALGTWVSYVSKLGKNPIDELQVKLS
AQKYDDAVVAKMIALSRYDVNSDFAADLGRSLQKKWKDSDNTESGVFHLLKLDDEKTGLLTNPVLEFWMRYVDSLGKDPYKL
LLLAINRKGINDAGLARMVGLAQKDRNNYWIGTNFESRVLDKWKQDGKSGNKLFTMLELDKEGDKVFDSPVWKIWTSYLNK
NEQEPNKIIYSVLRDRFDDSKLATLVSSSKEAENTVLNLWKTDGKTINQVFNLLKLDKTGDEIFERSAFSIWMAYVHKLNPDGLD
AFTLLEKRYGSKKLAAMLSRSLRGKHSADTKQLFSKLQELQFKKWMGENNNNPTAVHSKAESIGIAVAIDFQSYRIRPHHPYYM
DVF
>XP_008911008.1
MRFYYIVFAVTATLLASTNAVSTDAQHNQISQSTSEIVAATQIDVSVKRSLRSSKYIKEEEDTVDSLDDTEERRGGVLSKNLMKIV
KERNTPIAVNIAKLNPKQQEKIVDILYSQSRTLEYFAKKLGLRSAADTTHRNSPFFQAWSSYFLNGKKPKKIPEIWI
>XP_008908217.1
MLFYLNVLLVAIVLLACVDSASDNAASTATKCFPVANKSFLPTRRFLRAYATVHKDSEERVNVGNFALQEGVSKLAESARLKKFLL
KKKTGVEVLNSLKIGDDIAAALESSRLGTLTKYIAKFNKKNPDKTISLIETLTTRYGGSAVEKALASVERSTDSSPKVVDLVKQLRSE
QLTFWLHSDNSVDDVFKLLNSAYAGPEFFASPLFRILDDYIAKFNSEKQGQETLIKTLTRGFRGESNFVWLLAGAKQQTHDLRIV
NKAQELEEGLIRQWRHEKVEPVAIMKVLKLDNGAENALRSENLEMFDKYITLLSKNSQHNEVTLLGILTSKYGEANVAKAIAPAL
RREKANTELIAARLQQQQLKGWLENGMSPDDVFKLLWLYNGKRLVIAQQWDMLDTLDEFIMLYNRYNRAKPSDQTSLVRVL
ANSYGESKLAKLLVTRLEAPLFMSKAAKLQKAQFREWMDEGLEPASILSKIFKVTEADASNTQKLVASHFKRFYDTKNAAQPTFT
DPRRS
>XP_008894738.1
MHLHILVGLTIVIYFVADISATEPTGSMSSVNDEVDFYNVSKTRVSDTRFLRIVVDGNEEERTGLSVPPSEKLKSWLKSSNITPEKL
QKWLNKEKSPDIVFSRMQLTKAGDLLFYNSKFSTWLQYVDDLSKTSKEEVSAIKTLTARYGDSKLYGMIKSIKRFPEEKSLATRLE
EEQMQHWVNLRKDPDEIFQLFNLAENRRYIFRHPEFTTWVKYVDSLKAKHPKEPVSMTQTLTKYFSDKYLLELVKEAEVRKGSK
TVATKVEDDLLEFWLKDGKSPDDALVEVGLGMYRYFHLENPMFDTLAKYLRVYNSKYPDKKTTMIELLTKKFGDGDVSAILTEA
KLVDATTISSVGDVAEQREVCRTWVSYMNTIATNNPHDVHAIFSSLESQYVGRPLLQILEAAKQYPSMENTALGLQTKTIRSIFA
SGETPAVAFSQLVLDQAGDGLLSNPLFKNWMEYVKDFNKKNPEKQESWFQPLRIAYQWRIENMIARAKQNPTTVNIANTVE
RAWRKQWVEWNYAPSAAFKFFQLNRAYEKTLLSPEFKVWVKYLNYFNRQHPDKKETIIDGMKASYNDINILLILNAAKKDPNT
KKMVTYLQNELVNKWVVANEDPAVLRRRFSGTVENGEEMVQRYIKKREAMSATTS
>XP_008894082.1
MSSLQLFVLVVLGLCCAAASSLTNVDPTNIYLDTNVMVAADHKQGKRSLRQYTAYDLDEGDSEDEERGVNISEQLDGVLSKVD
DAVGKAGKAPSKLDALIMKNVDEFAKFADEHARLSKELFGKYPRANELSLSTLRQLEKIEKVREMDIKNGLKGNKVEPDGMRRK

IVPFPGMKIAPEQDLLSKVGRDTEHYKTVGGRALAAGIVTRTNGKGEHEILLISSSNPEKYEFLIPKGGVEKGEEVRKAAVREVVE
ESGVKADIVRELDKIKISEEKSIQPYLMKSDVVYDDWSESLRHRLWVPYKDAINLLDNRPQMLEMVKGAKKHIG
>XP_008910643.1
MPRYSIALFSVATVFVVSIASAVTAVDLSAVVSVKRNGIICKRLLRTHEAENENDERAGFGGLIQEGTSKVKDFITTKKYKSLVNRL
QLGDDFLDSVKSPQLERLNKLVTKFNRNKDQENQLSLIGPLSAKYGDDTVATMLVDLKKNPELAKLAKQLQDQQLTDWLKNG
KSGGDIFSLLKLKDDGNIGSQKLQVLEDYLTIVNTKKSTDETLLNVLTKGFGGESNLVPLLAKAGIDPLMVDKSKKLEAALLTKWR
DDNVPRLTVWYVSQNHPDHEISEIKKITAKYGEFAVAKALASAKDDQLMKDLAMKLQSLQLEIWLANRKSAANVFTLLQIKHD
GGSMKTWKMETLREYIQLFNSKTPQDKTKMFDVLRNGFGDGDGALARVVTKALTLADSPQMAAQYQSALFRRWVRSEIEPR
SIYARFLNVDEASANFLERSIVAQYKAYYDDVKNPPQAVTFHDPRRS
>XP_008910811.1
MRLNAILLVAAAALFASCDAVSTASQTKLSAMNPPNAVQSMGVAPYKRLLRSESQKQTDADDSEEEEEEEERALVKVGLLDDA
VKKVSGVDDAAAQKLAALKIKQQNEADDITFLAKFWMQQRKMPSDIEKLTQNPVVKKANLEFLALFLKQNPKQIRAEAARVQ
QRIDNADPKVRFLVSSWIFQNKKPEDISMLASNAVSKEAADLFNVQYVIRSLMSTK
>XP_008893649.1
MRPIYVLLLAVCIAIASCDAVSAFVTEQEGATAQRAAAVYRGTSGKRLLRSDDAATVDDDVNEEERLFGVSKLTEAAKKGKSKMI
DMHTKLKDQSLLGTFQLMKKGGFSDEAITSAWLTRGKSLDDIFDRWIRLGKSEWQAANNLLRQNKTPDDLYSVFAKRGMNSE
QIQTLWRSLKLDEDKLIALQKKFVPVN
>XP_008910638.1
MRRNSISLLTTLVFFLACVSSVSAETTAVNVPVNARLYATNQRHGHVFTNRQLKGYGTTTESDEERVGLGPLTGLVHAATSKIDD
FIAPAKYNYLVNNLRIGNSVEEALTSSQLKTLSQFVAKFRNKNPGKYISSIAPLTAKYGDDVVARALVQLERDGDMTDLVKQLRG
EQLMDWLRTGKSVDDVFNLLKLKEDGYKLLHSRKLRALEDYITLYNREKSTDETLVKVLATGFGGDGNLVALLEQAKKHVRSVY
KASSLDAALQTKWQSEKLPPMNVWSRLQFSDDVNEAISSGKLDMYYKYISENYRKSRGSVLERYIAKYGEVEVAKALVTAKGN
DATRKLATRLQKQQLEGWLATKKFPDDVFVLLKIKDDGILFTRSRKLKTLTTYIKMYNTHKKTQDQADIFRVVINGFGGDAAFAR
VVVKGVTMPDSPEMFRTAQNYEDALFKGWRQSQIEPKDVITKVFNKENGAASSLETEIAARYSEFYKRKTPAAS
>XP_008912262.1
MHLSSTLLAVSAFALLASANTLTIDSGKSVISTIQQPDSNKFIHTSKANESKRSLRLRETDHIRDDAPDEEERTGLDRAKMLSDKTY
RKQVFAQWLNNNYSSMTVFDILDVSNFEQYRRLYNKYAKLHKSSGNYP
>XP_008917463.1
MRLSRVLVVIAATFLATTDALSTNTGIHAVNVISHNGPSQRLLRSDYTTAEEDDDSEARALNLEKMKTMLDAGMSVDDYAFKL
KLTDKIAAAANSAKEMAKLAETHKFKKLLLYLNYVAEHT
>XP_008894979.1
MRLHYFLLVAAPMLLASTDFVAASTDSAHTKLSTAAESTHGVRMPARALTEGNDKRSLRISKTNDEDDEEENEARGIPVVSFYT
DEISARGLIRFLKKKSIISKARAKELKASDVSKEAIAEMYSNYVALATRPKRGEAIYSP
>XP_008893493.1
MRLPLVLLLAVCIAITSCAALSAFATEQETSVAQTAKRWGSPHSIAAAGDRDMSGKQLLRADNLLLVDNDVDAEERGLPAITKL
TDMVKKGKFAVGTKLSDKMLWVKYQKLLKQKLSDLDITGMWLKSGKSPDKIFDRWIRLDKSPRQAAKNLLNHGTTANDLYKV
LKKRNMDLETIRPIWRDLGLTENQLRVARQAASAL
>XP_008915662.1
MRLLLWVLLATLITFISSSHAVSAVADSDEPKVTQLTMKDIDIVTRLLFVEDGDAAKRFLRGNAKQDLTTANNDLDANDEERGLI
PSTLTNLITKAKNGWAKWKANALEKAFQHMMKLGETPTSLAKRLEIGGAAELRYEKLYEKYTAWWINYHTVAGT
>XP_008910745.1
MRVPSAILAALLLTYAASASTAGFPRSTVNNWHDTMRFLRTHEALNTEERGITVPNVEMLQGWLKKGLLSDEAVGLLSLGNKA
DNLLSGSLLNAWVSYIKVFNKENPTEKMQTISALTARFGDEALSTMIETAKRMPSTKDVANKIQTKQIQHWMKAGKTPNDVF
VLLELNSAKSILFDQPAVNTWLRYLDDFSASKPASQYSTIATLRTLYADDELAKMIVIASRNSKTSEAGKRVETELLRTWFNEMKT
PKDVSRNSKTSEAGKRVETELLRTWFNEMKTPKDVLRLLNARNQKKFSYASIWTKYDDLFTKVDPKFKTDMLEDWVKKGLITD
DTFRMLTLGNAADELLNGSLLSAWATYIKVFNQENPTQKMNLLAALTARFGDEAENPTQKMNLLAALTARFGDEAVAIMLET
AKKIRKTRSLAVSIQTEQIRHWLRTKKRPDDVFVLLKLETAKARLFDQPQLNTWVRYVDAFNNANPTRSTTLFSVLNARYYDEAL
AKMLVVAKSSGGSAGSLASRIQAEQTRYWLSVNRTPGAIFEMLQLETLGTNFLNHPIFTAWVKYTDDFRKKNLGTRLSTLTTLR
VYYSDATLAKLFTEARKVTKTAKIGRRLEAELLREWSLAVAPPALIFERLKLGNGGQKLFESPLFTMWTNYIAMFKKANPRYKDD
QLATLLRSYGRRELTLMLILAEKVPSTKDIATKLRGQLSGL
>XP_008916350.1
MRLFNMTLVVLAALLLAGGTAVSNADQASVLNVDVVHSSRILSGEDKSFLRRHPTELDEEERGGGANLFSALKQHRMKTDLDY
RLKVFARWKYYEKSIDDAKEHATKHLADAYEEFLKSKTRSGIEMNPLRIHRQD
>XP_008903809.1
MRLQKGLLLLFAGILFTRIHIASAESDAQFNTASVPPVPDRRLRKHNFELAKEGSDTEERGLNAEAAAKAFAIDAKKIDDVASKM
EIEMANVKYNWLKYLAHQKLRDFEGHSSQVAKNVAKYFSEWKASGYGATNVREEMVAAGITDEAAIQNAQKWFAIFAKKCR
VVAQDF

>XP_008891276.1
MRIHYAVRLLVFFLASANFVRASMENKNIASPNEISSNRAAAATRVLESHSAADIRKVDDIDEYEERGGLSKLVTLASKVNPSGT
NAVLLGLNKRPDRVFTLFGVGKKLDNKQEVFNWLRFTGKYQAKHGGKDSFPSSNILSLLRSGEKADAKLALLIQSVKQVRDIKEL
GRNMEKALYSEWFAKKLRPVHIRKLLADSRGSAPDVVKQIVENYAWRYVKLLGEKLTKEELEKLGPPLVVVQGLLKEK
>XP_008913167.1
MRLYSIVLTIPAVVYLSRVDSVSAADTTKAFTHSRSAVVDHQNTNQRYLRAYATTVKNGEERVFEALLNAAKAYKINTKLNAFLS
KMDDGVGVLNSLKLGNDVVSALTHKKLTQLTTYIAMFNENNLDKTISLIGVLTARYGDDAVAKSLAYLEKYAESPQVVELAKQL
RKDQLSSWLNDGKSVDHVFQLLKLGDEGVTVFANRKFTVLEDYITKFNSETNGHATLLNTLLKGFDGESNLVTILSHAKRDLATE
QLARKLEKELLGQWLQRDLEPEKVMMLLNLDNHVDVVLKSENLRTFEKYIAEFNGKNSNSQLSLLGMLTEKFGEVGVAKAIVS
AKENSNSAWVANKLQKQQLQGWLDNGMTVDDLFTHLKLKTDDTAVFGRSLNTLDDYVKLYNTANAKKDTLMGVLLREFGG
ESALANKLSQARVYTETHAKATKLLTMQFQKWKSGGLNPTNLVAKLSEKAEDGAPKLKKTVVNEFKKFHEGKKLQ
>XP_008912832.1
MRSMNVVQMFAVVVALVATSSAASNSSPTIASDRSSSELKFPFSAAEKTDNNEKHFLRSLKVPDKPVTDEEEERVINFSFLKNIQ
KKLPGTAAYKAAKAAKAAKEKAAIVDVLKMSDSRKDLLHSLFRQWDAEKKNHVNVADDVFKIMKDHGWTTKQANDIGVEYF
SFRSSLYV
>XP_008911080.1
MTTIARFRYLLLFVSAALFARIDTVSAAAETTLSKSAFHLSTQSLTAEANAASVFRALRALRTDAVSEGDEERAINIKSIPGLESLKKI
PGLDNLKKVSNLFKTKNTPGTYLKWAKKGKSPDYVFLKLKLDKTGYKLFQSADFNVWVAYTRMVSKTDADRAIFTSLLARYGDV
KLMRMAEAATKVDGTKEIATKLQKIQLDDWKYLGTSSDDVFRMMKLDDKADDLLTNPNFNTWAKYLEATKESTGVSTPMIN
TLRTHFADADLLKAFKAAQANPETKQLGLNLEASLNNIYRLRRMKNGQRRKPVAT
>XP_008901015.1
MRTTYVILVAAASILASTSDASAVKVPNQNQLPKEHPIDVAQVTEDKTSRFLRRREISDENEERSSLGKLATKLDDMATLSALRAV
KDKDLISAWQHLQQITSSWKNREAILEFIQLDQANRKKVLKLIVENAAKTKGIQ
>XP_008911081.1
MRLYDVVLAAATILLTAIDGISIATDANRNPVDLSATTRSFTQEQQNIPTKRSLRVYTTEAEDNDEERAINFKSLPGVDKLKTAWT
QNKLSKYLKKGKTGDEVFSKLKLDKAGDTLFENPKFHVWVKYVTDYNIKAGKNEISMIPTLTKHYGDDVLVKMLEIASKSSVVDT
RRVASSLQEQQVKYWRSLDLLPTDVFTRLRLGDKGKLDDMLTNPSFFALNKYLVDYNVRYSKSLTMVEVLKGGYGDEAIARML
QQTATKAKDVEIQKMAVRLQNQQFEHWRDIGLNSDDIFKELRLNKVGVTLENSNFVVWGKFLQNWSGENTTPFKSLWARYG
EKNLATMLVAPREQAGDSVIPFLQNQLVNQWLAMERTPADVFKMIGSSDGGKKLVGAYRWSYRNKFNAA
>XP_008904234.1
MRLDLIVLVLATCFLAPVESTSKATKQAYSAVIDAHAIRPARSLLAEADVVKADEERAGVGNAVEALAAGTSKLIDVAKLEKYLNK
KKTAVYVLNKLQFGDDVAGALKNTNLEALNTYIAMLNNKNSDEVITLIGTLSARYGDDAVAKALVTAERNAEKAPELAALAKQL
RSQQVFAWLDNGKSVDDVFNLLKLRGNDYEALTSRKLDVLEDYITKFNRENPDHKTNLVKTLTKGFGGETNLVGVLQRAKGDI
RTRTKAFQLEEGLIRQWVREKLEPARVMALLNLDNDVNGVLNSSNLKIFQKYLQEFNTDNLNSPVTLLTALTSKYDEGDVAKAII
SALRHENTPLEKLAVRLQNELLDGWMKRDMSVDDVFKLLKFNNDVSVVVSRNLETLKEYIRLYNRVKSQDETLAGVLARGFGG
ESNLAQILVGARSFPQLNAQAKKLQRAQFKEWNERMNVSPRRVLPKIFDVTETSATELQKRVVRDFKTYVKDQNAAANRIVEP
RRLRT
>XP_008904227.1
MRFTSVLLLLAVGFLECVDSTSGHSASKTLTDHLTVVTDTQPITLAHRFLRTQGVDAIEDRAGLGKVTDALKAHAKKLTDTLTEIL
RQKKTAAEVLNSLNLGDDVAGALKKSKLEVLKNYIERLNKKNPDKTISLVGTLSARYGDDEVPRAIVSAARRTDSALHVKELATQL
RSQQLRAWLDNGKSVDDVFKLLKLGDDGYEALTSRKLILLDDYIVEFNRANPGHKTTLLKTLTTGFGGESHLVTLLAAAKHDVRT
KAKATELENGLLRQWQRENLDPASVMKLLNLDNGVDRVLNNRNLETFEKYIAVFSKKNPENPTTFLGALTMKYEEGEVAKAIV
RNLETLEEYILVYNREKKVSETLIGALAKGFGGEKKLAEMLMRARTYPDSKINAIKVKNAQFRKWRDRGLNPVNVLTKVFSVEEA
GASRIQKRIVKEFTTYIERKNAAVHRITDPRRI
>XP_008915777.1
MRLAYIFALGIAGTLHDSSSAFPATIANDKAMIDNEAPPNPIKMDGYRLLRRVDNDEQEIEEERTLNLGDVLKKLNPVKAVKKAK
EKVQDATDKITEPNWKDLVVHLAVRGDDKG
