# Supplementary material for: Short Linear Motifs (SLiMs) in “Core” RxLR Effectors of Phytophthora parasitica var. nicotianae: a Case of PpRxLR1 Effector
Source: Microbiol Spectr. 2022 Apr 11;10(2):e01774-21. doi: 10.1128/spectrum.01774-21 (PMC9045269; doi:10.1128/spectrum.01774-21)
Supplement: SUPPLEMENTAL FILE 3 — Supplemental material. Download supplemental_file_3.pdf, PDF file, 0.28 MB [file supplemental_file_3.pdf]

Supplementary figures

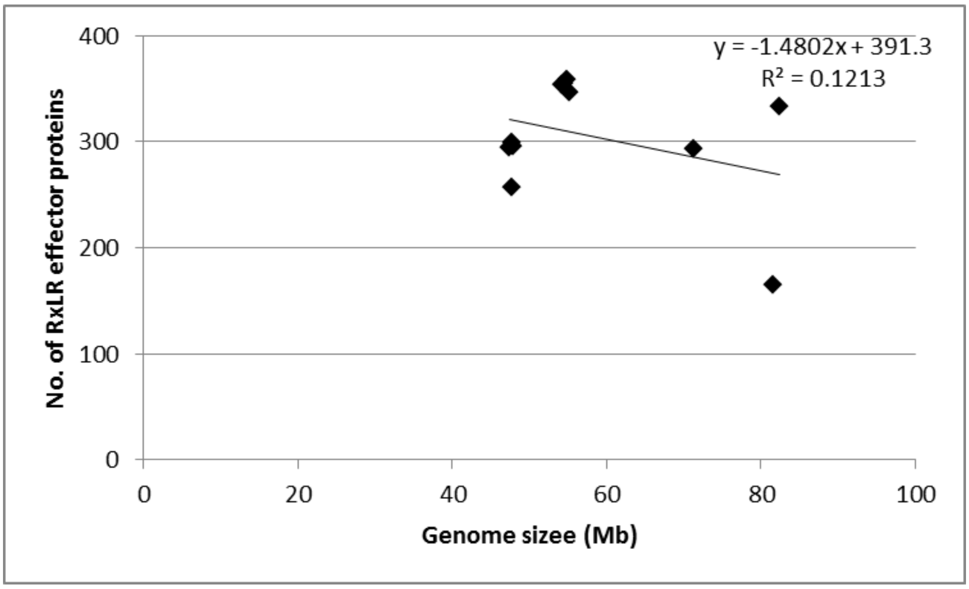

Figure 1: The relationship between genome size and secreted number of RxLR effector proteins in *P. parasitica.* Insignificant association was recorded (R^2=0.12;p=0.294)

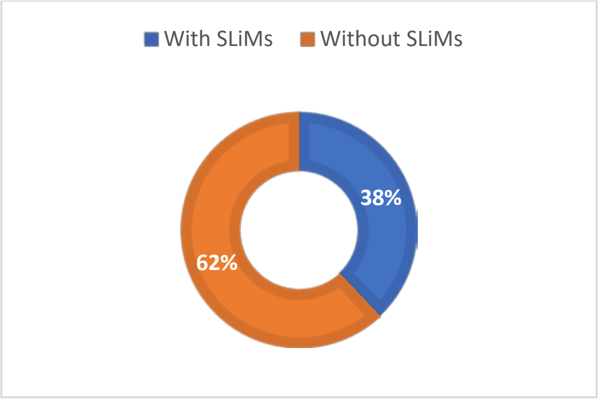

Figure 2: Percentage of CREs with and without SLiMs. More than half of the predicted CREs (62%) were shown not to encode known SLiMs while 38% of it encoded ELM-associated SLiMs

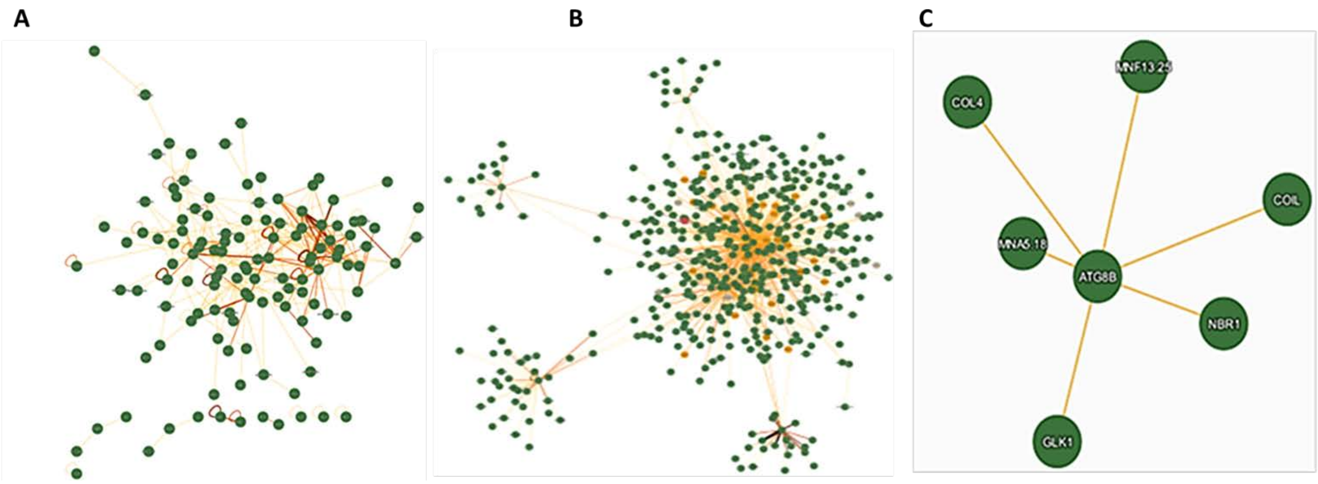

Figure 3: Fuzzy interaction network of PpRxLR1 (A), PexRD54 (B) and *Arabidopsis* proteins using IntAct on cytoskape. PexRD54 showed the potential to interact with , ubiquitin-like protein ATG8 (at the centre) that could potentially interacts with six other proteins (C).

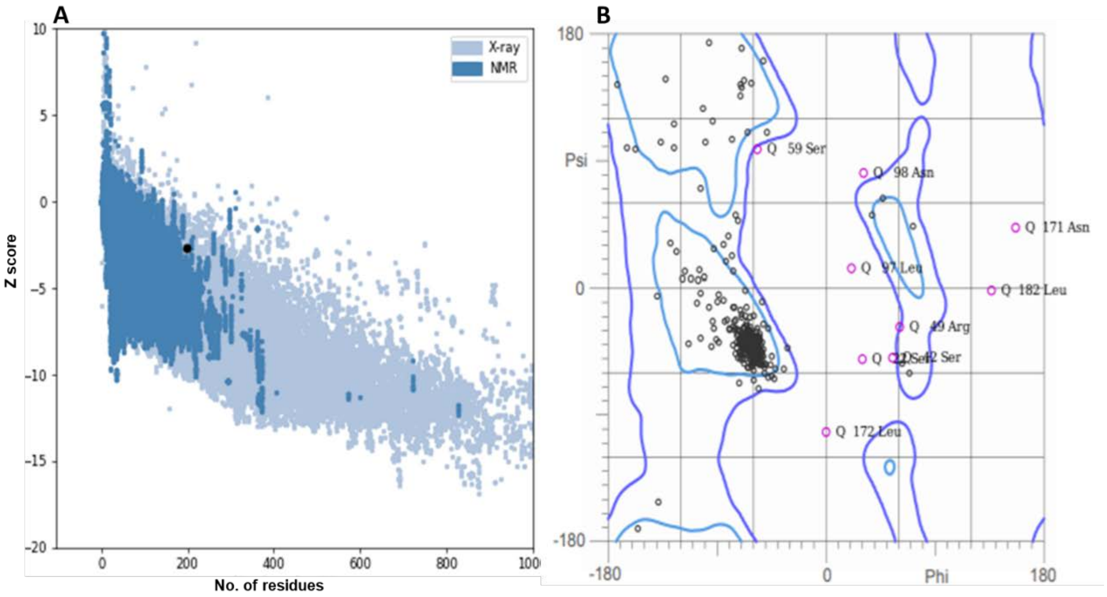

Figure 4: PpRxLR1 *in silico* predicted structure validation. The predicted structure was validated using ProSA Web and molprobity. (A) ProSA Web plot showing Z-score of the predicted structure −2.68 of PpRxLR1 (black dot) relative to Z-scores of similar sized protein structures solved using NMR and X-ray crystallography. The z-score indicates overall model quality. Its value is displayed in a plot that contains the z-scores of all experimentally determined protein chains in current PDB. Therefore, the quality of our predicted model was rated as good since the z score falls within this zone. (B) Ramachandran plot obtained from MolProbity showing 88.7% of residues lie in the most favoured region, while 3.19 % of residues were in the outlier region.

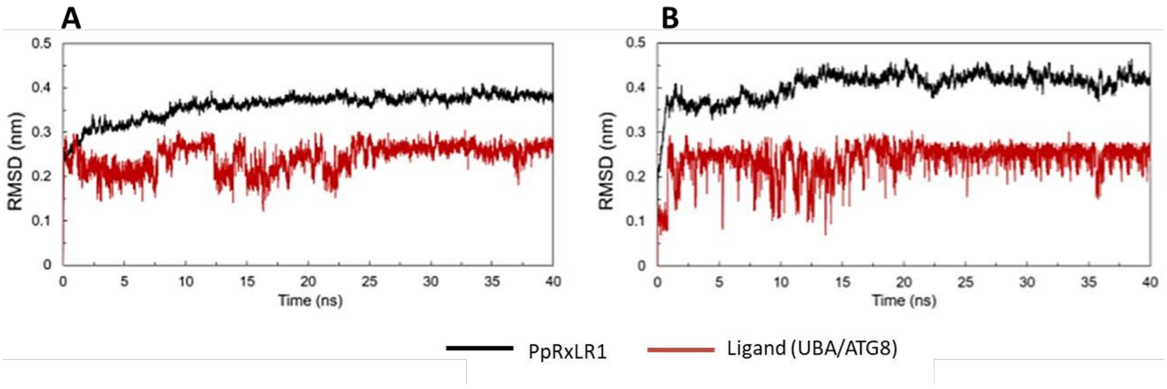

Figure 5: RMSD of the backbone Cα atoms of PpRxLR1 effector protein in complex with ligand molecules: UBA (A) and ATG8 (B). RMSD is one of the critical parameters to analyze a protein–ligand complex. It characterizes the overall conformational stability in a dynamic state during the simulation. The system is equilibrated and stabilized when it obtains low levels of RMSD with consistent fluctuations for the entire simulation; on the other hand, higher fluctuations indicate low stability.
